# *Testing Biochemistry* Revisited: How *In Vivo* Metabolism Can Be Understood from *In Vitro* Enzyme Kinetics

**DOI:** 10.1371/journal.pcbi.1002483

**Published:** 2012-04-26

**Authors:** Karen van Eunen, José A. L. Kiewiet, Hans V. Westerhoff, Barbara M. Bakker

**Affiliations:** 1 Department of Molecular Cell Physiology, VU University Amsterdam, Amsterdam, The Netherlands; 2 Kluyver Centre for Genomics of Industrial Fermentation, Delft, The Netherlands; 3 Department of Pediatrics, Center for Liver, Digestive and Metabolic Diseases, University Medical Center Groningen, University of Groningen, Groningen, The Netherlands; 4 Manchester Centre for Integrative Systems Biology, Manchester Interdisciplinary BioCentre, The University of Manchester, Manchester, United Kingdom; 5 Synthetic Systems Biology, Netherlands Institute for Systems Biology, University of Amsterdam, Amsterdam, The Netherlands; Medical College of Wisconsin, United States of America

## Abstract

A decade ago, a team of biochemists including two of us, modeled yeast glycolysis and showed that one of the most studied biochemical pathways could not be quite understood in terms of the kinetic properties of the constituent enzymes as measured in cell extract. Moreover, when the same model was later applied to different experimental steady-state conditions, it often exhibited unrestrained metabolite accumulation.

Here we resolve this issue by showing that the results of such *ab initio* modeling are improved substantially by *(i)* including appropriate allosteric regulation and *(ii)* measuring the enzyme kinetic parameters under conditions that resemble the intracellular environment. The following modifications proved crucial: *(i)* implementation of allosteric regulation of hexokinase and pyruvate kinase, *(ii)* implementation of *V_max_* values measured under conditions that resembled the yeast cytosol, and *(iii)* redetermination of the kinetic parameters of glyceraldehyde-3-phosphate dehydrogenase under physiological conditions.

Model predictions and experiments were compared under five different conditions of yeast growth and starvation. When either the original model was used (which lacked important allosteric regulation), or the enzyme parameters were measured under conditions that were, as usual, optimal for high enzyme activity, fructose 1,6-bisphosphate and some other glycolytic intermediates tended to accumulate to unrealistically high concentrations. Combining all adjustments yielded an accurate correspondence between model and experiments for all five steady-state and dynamic conditions. This enhances our understanding of *in vivo* metabolism in terms of *in vitro* biochemistry.

## Author Summary

Baker's yeast is widely applied in modern biotechnology, for instance for production of heterologous protein or biofuel. For such applications a thorough understanding of the central energy metabolism of the bug is crucial. Nevertheless, even for this well-known organism, attempts to build models *ab initio*, based on independently measured characteristics of the catalysts (the enzymes), seldom gives reliable results. A key problem in this field is that enzyme characteristics are often studied under non-physiological conditions that do not resemble the environment inside the cell. In this study we measured the enzyme characteristics under physiological conditions and assembled the results into a computational model of yeast energy metabolism. We show that this simple trick greatly improves the predictive value of the computational model. This allowed us to predict correctly how yeast cells adapt to nitrogen starvation, an industrially relevant situation, in which remodeling of the proteome strongly affects cellular energy metabolism.

## Introduction

Molecular biology is based on the paradigm that living organisms can eventually be understood in terms of physics, chemistry and organization. Indeed, biochemistry and molecular biology have furnished impressive examples where the precise mechanism of action of individual macromolecules, such as DNA and the proton-translocating ATPase was elucidated. At this stage, however, no functional *network* of macromolecules is understood in the sense of physics and chemistry, such that a computational model made precise predictions that have been validated by the corresponding experiments.

The best-researched biological network is the breakdown of glucose. The enzymes of yeast glycolysis have been examined qualitatively and quantitatively for almost a century. Since the 1960s many kinetic computer models of yeast glycolysis have been constructed. The early models focused on the mechanisms underlying oscillations in yeast cultures and cell extracts, and were not much concerned with quantitative precision [1], [2], [3], [4], [5]. Developments in Metabolic Control Analysis (MCA) inspired the construction of a new generation of models to study the distribution of flux control in glycolysis. The aim of these models was primarily to amplify or redirect the flux through glycolysis [6], [7], [8], [9]. Two more recent kinetic models zoomed in on the role of detailed enzyme kinetics [10], [11], [12]. The model of Rizzi *et al.* [11] implemented published kinetic mechanisms and affinity constants and fitted the *V_max_* values to the dynamic response of yeast after addition of excess glucose. Hynne *et al.* [10] modeled the dynamic characteristics of oscillating yeast suspensions to estimate not only the enzyme capacities (*V_max_*) but also their affinity constants (*K_m_*) [10]. Both these approaches used the modeling for estimation of the *in vivo* values of kinetic parameters.

Heijnen and colleagues have argued that the discrepancy between *in vitro* and *in vivo* enzyme kinetics precludes reliable modeling based on detailed enzyme kinetic equations [13]. To overcome this problem they proposed the so-called lin-log approach [13], a simplified kinetic description that is closely related to mosaic non-equilibrium thermodynamics (MNET) [14]. The lin-log kinetic framework resulted in accurate model predictions with fewer parameters to be fitted [15], [16], [17]. This ‘minimalist’ approach demonstrated the importance of the feedback and feed forward loops for glycolytic dynamics [18], [19].

Teusink *et al.* was the first to actually evaluate to what extent biochemical knowledge obtained from *in vitro* studies could be used to predict the glycolytic flux and the concentrations of glycolytic intermediates in yeast [12]. They concluded that the *in vitro* kinetics did not quite describe the *in vivo* activity for all of the glycolytic enzymes satisfactorily. In order to obtain a satisfactory description, they had to displace the kinetic parameter values away from their *in vitro* magnitudes, albeit only in a few cases far beyond the standard error of the means.

The model of Teusink *et al.* [12] was initially only tested for one experimental steady state. Implementation of *V_max_* values from cultures grown under other conditions into the Teusink *et al.* model, has not been very successful. Often the model fails to reach a steady state and accumulate intermediate metabolites while the real yeast cultures do reach such a steady state (S. Rossell, personal communication; and this study). Reijenga *et al.* [20] used the model by Teusink *et al.* [12] to study spontaneous oscillations. The predicted frequency of the oscillations was close to experimental findings, but the concentrations of the oscillating metabolites predicted by the model did not match the experimental counter parts [20].

The observed discrepancies between experiments and models could in principle be caused by various regulatory mechanisms, such as enzyme-enzyme interactions, channeling and rapid posttranslational modifications. Another obvious reason might be that, until recently, enzyme kinetic parameters were most often determined under non-physiological conditions that had been optimized for high enzyme activity (but see [12]). This was warranted in view of the original goal of such studies to elucidate catalytic mechanisms, but does not fit the newer goal of understanding the *in vivo* dynamics of metabolic pathways. So far enzyme kinetics have hardly been studied under conditions that resemble the intracellular environment. Recently, we therefore developed an assay medium that should resemble the yeast cytosol [21]. A substantial number of enzyme activities measured in the *in vivo*-like medium differed substantially from the activities in the ‘optimal’ non-physiological media [21]. These differences might provide a way out of the above impasse.

Here we therefore revisit the question whether *in vitro* enzyme kinetics can be used to predict *in vivo* yeast glycolysis. To this end the computer model of yeast glycolysis by Teusink *et al.* [12] was revised by including *V_max_* values and values of parameters that were measured in the new *in vivo*-like assay medium. In addition, hitherto missing but known allosteric regulators were implemented in the model. We show that if both innovations are combined, accurate model predictions are obtained. In contrast to earlier studies, a single model could be used to describe five different experimental situations.

## Results

### Summary of the experimental data used in the glycolytic model

We validated the revised model of yeast glycolysis by comparing the model predictions to independently measured fluxes and metabolite concentrations in *Saccharomyces cerevisiae* before and after nitrogen starvation. We studied: *(i)* non-starved cells from a glucose-limited chemostat culture grown under respiratory conditions (dilution rate D = 0.1 h^−1^), *(ii)* cells grown under these respiratory conditions and subsequently starved for nitrogen during 4 h, *(iii)* non-starved cells grown under respirofermentative conditions (glucose-limited chemostat, D = 0.35 h^−1^) and *(iv)* 4 h nitrogen-starved cells derived from this respirofermentative culture. From each of the four cultures yeast cells were harvested to measure *(i)* the maximal glycolytic flux and the intracellular metabolite concentrations in an off-line assay under anaerobic glucose-excess conditions (fermentative capacity [22], [23]), *(ii)* the *V_max_* of the glycolytic and fermentative enzymes, and *(iii)* the kinetic parameters of glucose transport across the plasma membrane. The fluxes and metabolite concentrations have already been reported in [23] and are taken from there. The *V_max_* values presented in [23] had been measured under assay conditions that were optimal for each enzyme and that do not resemble the *in vivo* conditions (Table S5). We refer to these as ‘*V_max_* optimized assays’ to distinguish them from the *V_max_* values that were measured for the present study in the same samples under *in vivo*-like conditions (this study; Table S4). In addition, we have redetermined all kinetic parameters of glyceraldehyde-3-phosphate dehydrogenase under *in vivo*-like conditions and the kinetics of glucose transport in a 5-seconds ^14^C-glucose uptake assay (Table S4). Under both growth conditions the transport capacity decreased and the *K_m_* for glucose increased upon 4 h nitrogen starvation.

Input data for the model were: *(i)* fluxes to the side-branches that were fixed in the model (Table 1); *(ii)* external metabolite concentrations that were fixed (Table 1); *(iii) V_max_* values of the enzymes (Table S4 and S5 for *in vivo*-like and *V_max_* optimized assays, respectively); and *(iv)* other kinetic parameters of the enzymes and the glucose transporter (mostly taken from Teusink *et al.* [12]; as specified in Table S1); glucose transport this study (Table S4), and glyceraldehyde-3-phosphate dehydrogenase as indicated in the text).

**Table 1:** Measured fluxes into the side branches and concentrations of allosteric regulators and adenine nucleotides.

	D = 0.1 h^−1^ Non-starved	D = 0.1 h^−1^ 4 h N-starved	D = 0.35 h^−1^ Non-starved	D = 0.35 h^−1^ 4 h N-starved
***Fluxes***				
*Trehalose*	−2.1	−2.2	1.0	−1.9
*Glycerol*	17.5	24.9	21.3	21.5
*Succinate*	0.9	0	0.9	0.2
***Regulators***				
*ATP*	5.00	3.92	4.29	4.70
*ADP*	1.00	0.81	1.29	1.09
*AMP*	0.30	0.25	0.44	0.37
*Trehalose 6-phosphate*	2.20	0.36	3.52	0.59
*Fructose 2,6-bisphosphate*	0.014	0.009	0.003	0.006

The fluxes to (if positive)/from (if negative) trehalose, to glycerol and to succinate are given in mM.min^−1^ under the growth and starvation conditions studied. These fluxes and metabolite concentrations were subsequently used as fixed parameters in the model described in this study. Positive values indicate fluxes away from glycolysis. Data from [23].

**Figure 1:**
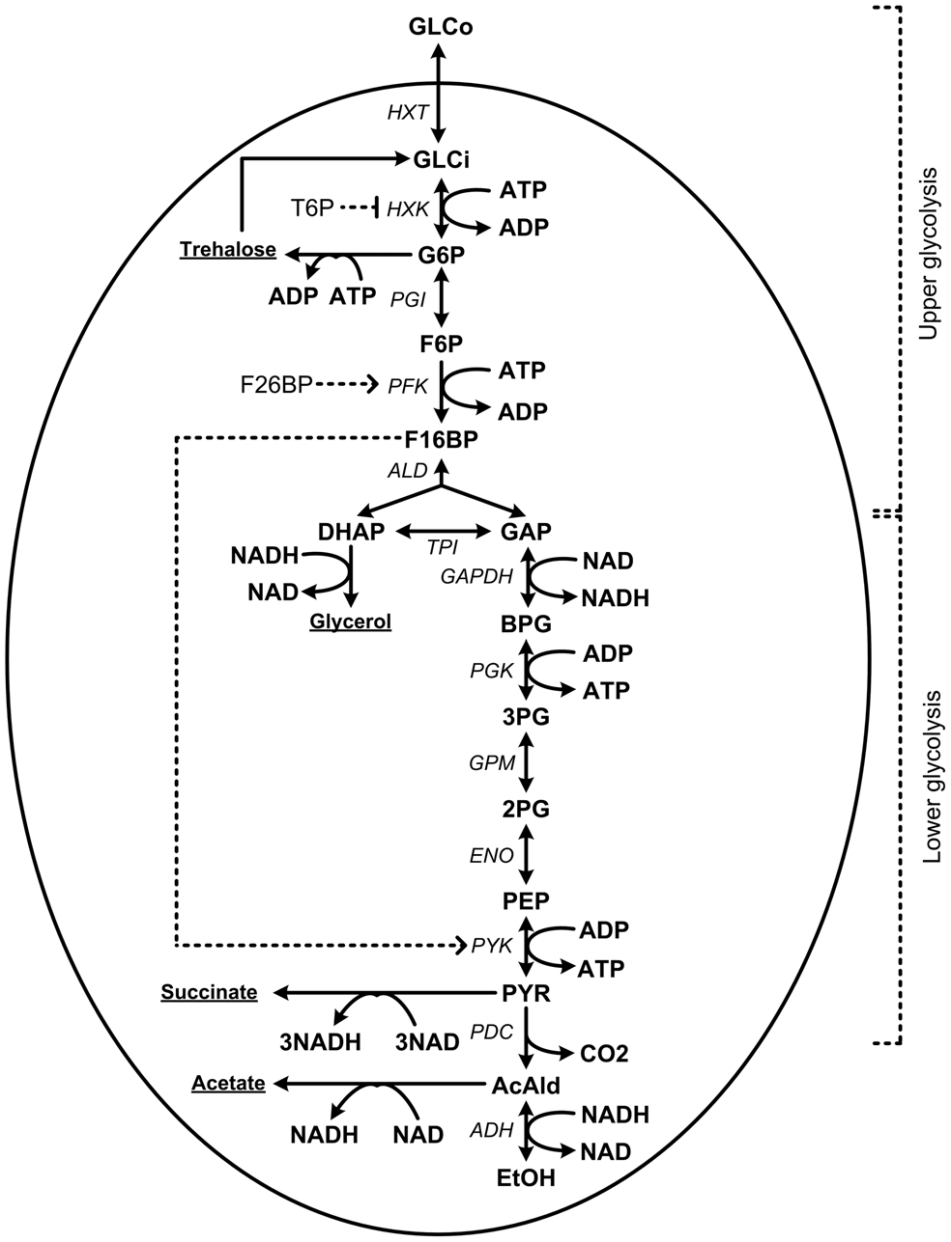
The glycolytic and fermentative pathway as they were modeled in this study. Metabolites are depicted in bold face, allosteric regulators in regular, enzymes in italics and branching pathways underlined. GLCo: extracellular glucose, GLCi: intracellular glucose, G6P: glucose 6-phosphate, F6P: fructose 6-phosphate, F16BP: fructose 1,6-bisphosphate, DHAP: dihydroxyacetone phosphate, GAP: glyceraldehyde 3-phosphate, BPG: 1,3-bisphosphoglycerate, 3PG: 3-phosphoglycerate, 2PG: 2-phosphoglycerate, PEP: phosphoenolpyruvate, PYR: pyruvate, ACE: acetaldehyde, EtOH: ethanol, HXT: hexose transport, HXK: hexokinase (EC 2.7.1.1), PGI: phosphoglucose isomerase (EC 5.3.1.9), PFK: phosphofructokinase (EC 2.7.1.11), ALD: aldolase (EC 4.1.2.13), TPI: triose-phosphate isomerase (EC 5.3.1.1), GAPDH: glyceraldehyde-3-phosphate dehydrogenase (EC 1.2.1.12), PGK: 3-phosphoglycerate kinase (EC 2.7.2.3), GPM: phosphoglycerate mutase (EC 5.4.2.1), ENO: enolase (EC 4.2.1.11), PYK: pyruvate kinase (EC 2.7.1.40), PDC: pyruvate decarboxylase (EC 4.1.1.1), ADH: alcohol dehydrogenase (EC 1.1.1.1).

Model predictions were compared to *(i)* the independently measured fluxes through *hexose transport*-*hexokinase*, *phosphoglucose isomerase-aldolase* and *glyceraldehyde-3-phosphate dehydrogenase-alcohol dehydrogenase* (see Figure 1); and *(ii)* the independently measured glycolytic metabolite concentrations.

### Glycolysis model

Our aim was to investigate whether the use of *in vivo*-like enzyme assays rather than *V_max_* optimized assays would bring model predictions closer to measured metabolite concentrations and fluxes. The analysis was limited to glycolysis. Therefore the fluxes to side branches as well as boundary metabolites, such as ATP were fixed (Table 1). In its present form, the model is therefore not suitable to study dynamics in which ATP is involved, like glycolytic oscillations. Furthermore, allosteric regulators that were missing in the Teusink *et al.* [12] model, were added. A full set of modifications is listed in Materials and Methods.

**Figure 2:**
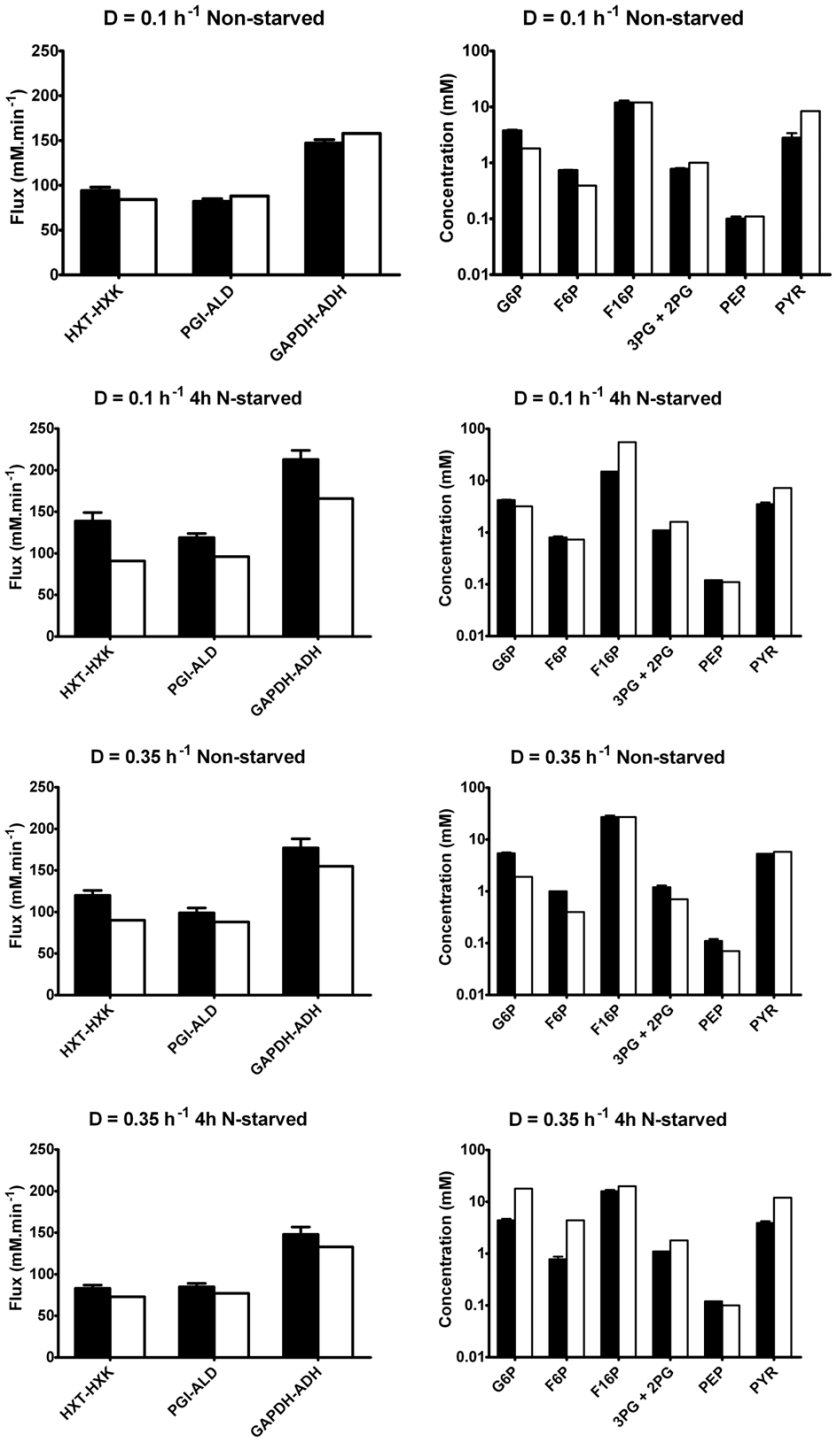
Predictions from the model of yeast glycolysis compared to experimental data from [23]. Black bars represent the experimental (± SEM) data and white bars represent model predictions. The *K_i_* of HXK for T6P was 0.2 mM for the non-starved cells from the respirofermentative culture (D = 0.35 h^−1^) and 0.04 mM for the other three conditions. Abbreviations as in Figure 1.

The new model reached a steady state close to the experimentally determined steady state for all conditions studied (see Figure 2). To achieve this, two additional assumptions were needed. First, the expression of the two hexokinase isoenzymes, *hexokinase 1* and *hexokinase 2* was not known. These isoenzymes have a different *K_i_* towards T6P (0.2 mM for *hexokinase 1* and 0.04 mM for *hexokinase 2*), which had a substantial effect on both the flux and the intermediate metabolite concentrations. In Figure 2 the *K_i_* that fitted best was chosen for each condition (model results for both *K_i_* values are shown in Tables S7, S8, S9, S10). Second, the kinetic parameters of glyceraldehyde-3-phosphate dehydrogenase were redetermined under *in vivo*-like conditions. Especially the *K_m_* values for glyceraldehyde 3-phosphate and NAD^+^ differed from those in the Teusink *et al.* [12] model *and* affected the model predictions. The measured *K_m_* for NAD^+^ was similar between the four conditions (2.84±0.15 mM as compared to 0.09 in the Teusink *et al.* [12] model). The *K_m_* for glyceraldehyde 3-phosphate differed however (0.39 at D = 0.35 h^−1^ as compared to 1.68±0.70 mM for the other three conditions). In Figure 2 we used the *K_m_* for NAD^+^ of 2.84 mM and for glyceraldehyde 3-phosphate of 0.39 mM under all conditions. Tables S7, S8, S9, S10 report the full set of results with the glyceraldehyde-3-phosphate dehydrogenase parameters specific for each condition.

Thus we obtained a single model that can describe four different steady-state conditions when the result of altered gene expression – measured at the level of *V_max_* – is implemented.

### The relative contribution of the in vivo-like V_max_ values and the allosteric regulation

**Figure 3:**
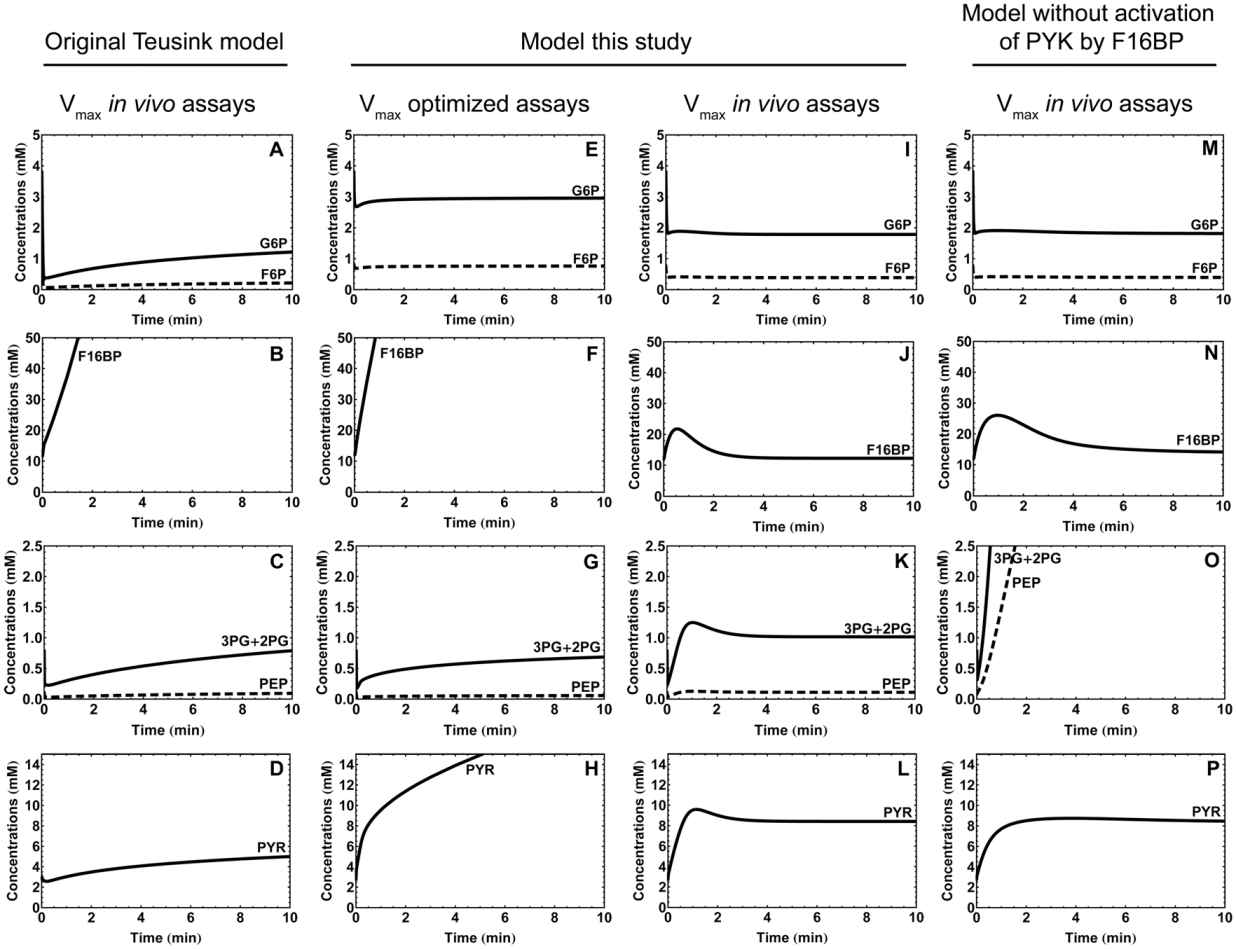
The comparison of the model results of yeast glycolysis obtained with the original model of Teusink *et al.* [**12**] (panel A–D), the model developed in this study (panel E–L) and the latter model without activation of pyruvate kinase by fructose 1,6-bisphosphate (panel M–P; see text for model description). The data used in these simulations were from the non-starved cells of the glucose-limited respiratory culture (D = 0.1 h^−1^). The *V_max_* values were measured in either the *in vivo*-like assay medium (panel A–D and I–P) or in the assay medium optimized for each enzyme (panel E–H). In the former case also the other glyceraldehyde-3-phosphate dehydrogenase parameters measured under *in vivo*-like conditions were used; in the latter case the original glyceraldehyde-3-phosphate dehydrogenase parameters from Teusink *et al.* [12]. The concentrations at time zero equal the measured intracellular concentrations. Abbreviations as in Figure 1.

Subsequently, we asked whether the good fit between model and experiment could have been achieved by implementing either the allosteric regulations alone or the *in vivo*-like *V_max_* values alone. The dynamic behavior of the new model was evaluated *(i)* with the *in vivo*-like *V_max_* values implemented in the original Teusink *et al.* [12] model (hence without the allosteric regulation; Figure 3A–D); *(ii)* with the *V_max_* values measured under conditions that were optimized for each enzyme, but in the new model (with allosteric regulation but without *in vivo*-like enzyme activities; Figure 3E–H); and with the *in vivo*-like enzyme activities implemented in the new model (with allosteric regulation; Figure 3I–L). The comparison was made for the non-starved cells from the respiratory culture (D = 0.1 h^−1^). The metabolite concentrations measured under these conditions were taken as the initial concentrations. In case of a good fit, the calculated concentrations should therefore stabilize at their initial values. In all cases, the concentrations of glucose 6-phosphate and fructose 6-phosphate dropped rapidly and then stabilized. Fructose 1,6-bisphosphate, however, increased continuously up to very high levels when either the allosteric regulation was lacking (Figure 3A–D) or when the *V_max_* values had been measured under non-physiological conditions (Figure 3E–H). Apparently, the lower part of glycolysis failed to keep up with the flux through the upper part of glycolysis (Figure 1). This resembles the ‘turbo’ phenotype described earlier [24], which has been attributed to a lack of product inhibition of hexokinase and phosphofructokinase. In earlier studies, however, not only fructose 1,6-bisphosphate accumulated, but also glucose 6-phosphate and fructose 6-phosphate [25]. Only when both the allosteric regulation and the *in vivo*-like *V_max_* values were implemented (Figure 3I–L) a steady state was obtained.

Teusink *et al.* [12] attributed the turbo phenotype primarily to a compromised feedback regulation of hexokinase. To test the role of the positive feedforward by fructose 1,6-bisphosphate to pyruvate kinase, this regulation was removed and otherwise the model and its parameters were kept as in Figure 3I–L. The result (Figure 3M–P) demonstrates that the feedforward regulation of pyruvate kinase hardly affects fructose 1,6-bisphosphate itself, but it is required for homeostatic regulation of the downstream metabolites 3-phosphoglycerate, 2-phosphoglycerate and phosphoenolpyruvate.

### Simulation of the response to an upshift of the extracellular glucose concentration

**Figure 4:**
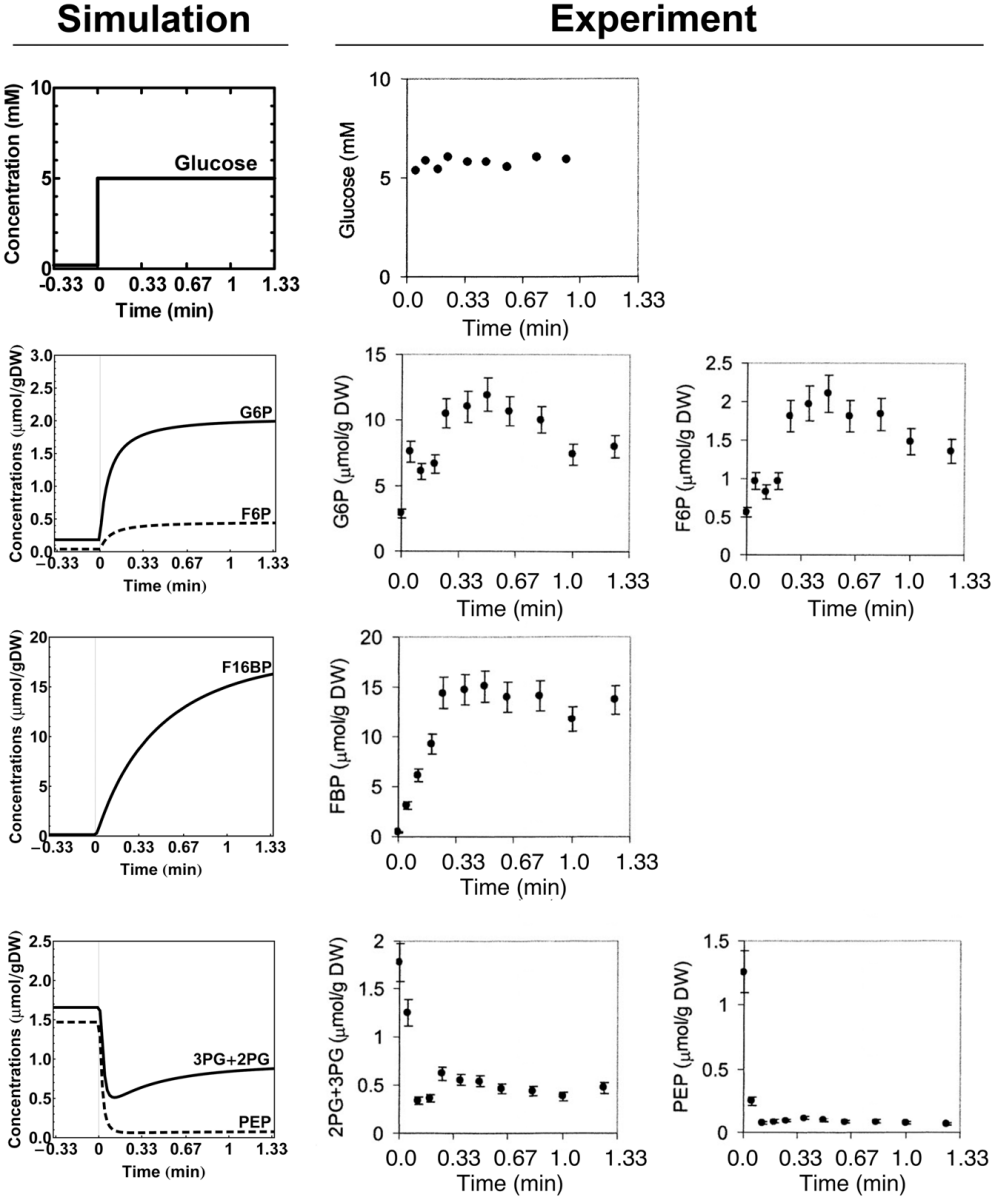
Simulation of a sudden upshift of the extracellular glucose concentration starting from a steady-state, aerobic, glucose-limited chemostat culture at a dilution rate of 0.1 h^−1^. The ATP concentration was decreased by 50% at the onset of the glucose upshift, maintaining it constant thereafter. Experimental data are taken from [26] with permission. For details of the simulation, see text. A complete list of parameters values used is given in Table S6 of the supporting information. All other parameters were kept the same as in the non‐starved cells from the respiratory culture (D=0.1 h^−1^).

So far we discussed steady-state conditions. A dynamic response, however, contains much more information. Intracellular metabolite concentrations measured during the dynamic response of *S. cerevisiae* to a glucose pulse have been reported [26]. The starting conditions of the experiment are close to the respiratory growth condition (D = 0.1 h^−1^) applied in the present study and therefore we used our dataset to model the response to addition of glucose. There are two differences between the experiment and the model simulation. First, in the experiment a single dose of glucose was added at time zero, while the modeled increase of glucose was sustained. However, within the time frame of the experiment the extracellular glucose concentration did not decrease significantly (most upper graphs in Figure 4). Second, the starting condition of the experiment was a steady-state, glucose-limited chemostat at a dilution rate of 0.05 h^−1^ while the simulation was based on the *V_max_* data obtained at 0.1 h^−1^.

The model version of the non-starved respiratory cells was used with a few modifications to meet the specific experimental setting. The residual extracellular glucose concentration prior to addition of glucose was approximately 0.2 mM [27]. Starting from a steady state at 0.2 mM glucose, an upshift to 5 mM glucose at time zero was simulated. The intracellular ATP, ADP and AMP ([28]; measured in a culture at D of 0.1 h^−1^) and trehalose 6-phosphate ([27] measured in a culture at D of 0.03 h^−1^) were also implemented in the model. In agreement with experiments [26], [29], we decreased the (externally imposed) ATP concentration by 50% at the onset of the glucose upshift, maintaining it constant thereafter. Since there was no data available for the intracellular concentration of F26BP, we used 0.014 mM, as measured at a dilution rate of 0.1 h^−1^. The model was evaluated at a *K_i_* value of 0.04 mM for the inhibition of hexokinase by trehalose 6-phosphate, like the steady-state calculations for the corresponding culture at D = 0.1 h^−1^ (Figure 2).

Figure 4 shows that the dynamic simulation mimicked the experiment surprisingly well [26]. The upshift of the glucose concentration caused an increase of the concentrations of glucose 6-phosphate, fructose 6-phosphate, and fructose 1,6-bisphosphate, while the concentrations of 3-phosphoglycerate+2-phosphoglycerate and phosphoenolpyruvate decreased and then rose slightly to reach a new and different steady state.

### Control and regulation of glycolysis during nitrogen starvation

**Figure 5:**
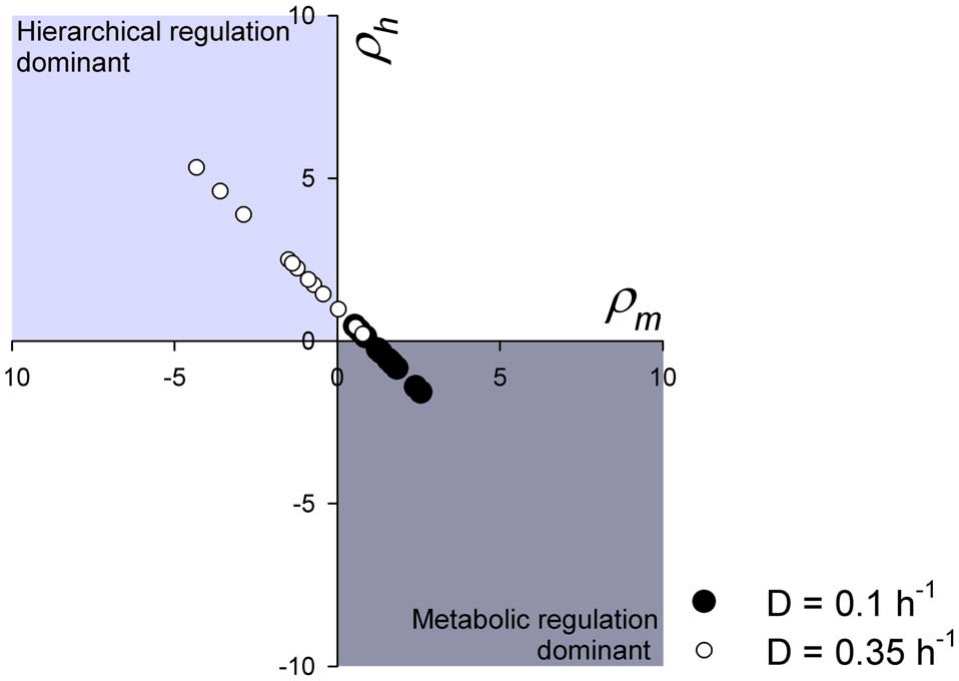
Distribution of the regulation coefficients of the various glycolytic enzymes after 4 h nitrogen starvation. The hierarchical regulation coefficient *ρ_h_* reflects regulation of the flux by altered *V_max_* through the entire gene-expression cascade. The metabolic regulation coefficient *ρ_m_* reflects regulation through interaction of the enzyme with altered metabolite concentrations. These values are based on the changes in the *in vivo*-like *V_max_* values, experimentally determined changes in fluxes and the summation law for regulation (*ρ_h_*+*ρ_m_* = 1). See [22], [23] for an elaborate description of how to determine these regulation coefficients. Closed circles: D = 0.1 h^−1^, Open circles: D = 0.35 h^−1^. Each point represents regulation of the flux through a different glycolytic enzyme.

In previous studies we analyzed the distribution of regulation between gene expression and metabolism upon nitrogen starvation of the same cultures as studied here [22], [23]. The upregulation of the flux through glycolytic enzymes upon nitrogen starvation of the respiratory culture (D = 0.1 h^−1^) was predominantly regulated by interaction of the enzymes with metabolites. In contrast, the downregulation of the flux upon nitrogen starvation of the respirofermentative culture (D = 0.35 h^−1^) was mostly regulated by gene expression. If we use the *in vivo*-like *V_max_* assay on which the present paper is built, we obtain qualitatively the same results as in the previous study (Figure 5 and Table S16) [23]. In this analysis the metabolic regulation reflects the cumulative effect of changes in metabolite concentrations on the flux through a specific enzyme. Often these metabolites counteract each other [23]. We therefore wondered if we could use the glycolysis model to relate the observed flux to measured *V_max_* values (gene-expression regulation) and metabolite concentrations.

At D = 0.35 h^−1^ the glycolytic flux (when calculated at the level of phosphoglucose isomerase-alcohol dehydrogenase) decreased by 16% upon nitrogen starvation. This was reproduced by the model (14% decreased flux). Under these conditions the highest flux control was exerted by the glucose transporter (flux control coefficient 0.5 for the flux through upper glycolysis and 0.6 through lower glycolysis; Table S14 and S15). At the measured 52% decrease of the *V_max_* of the transporter (Table S4) this should lead to a 26% decrease of the flux. Most of the remaining control was exerted by hexokinase (0.5–0.6 before starvation), but since the *V_max_* of hexokinase was down-regulated by only 12% this contributes only 6% flux reduction. Since other enzymes exert hardly any control, the changes in their *V_max_* do not contribute to the decrease of the flux. The fact that the flux through phosphoglucose isomerase altered not 26 but only 16% is at least partly due to the reversal of the trehalose flux from trehalose production in the non-starved cells to glucose-6-phosphate production the starved cells.

Under this condition (D = 0.35 h^−1^) metabolic regulation mostly counteracts gene-expression or *V_max_* regulation, with the highest negative metabolic regulation coefficient for phosphofructokinase (*ρ_m_* = −3.4). When we calculate the metabolic regulation based on the changes in the measured metabolites and the rate equation used in the model, we do not find this negative metabolic regulation coefficient back. However, when we used the modeled values of the metabolite concentrations, the regulation coefficient was −3.8. This can be explained from the discrepancy between the measured and modeled concentration of fructose 6-phosphate: in the experiment it declined slightly, but in the model it increased 11-fold from 0.4 to 4.4 mM thus counteracting the decrease of the flux through phosphofructokinase. Also for other enzymes with strong metabolic regulation, *i.e.* hexokinase, phosphoglucose isomerase, pyruvate kinase and pyruvate decarboxylase, the metabolic regulation was only reproduced with the modeled concentrations. Again this was caused by (sometimes subtle) discrepancies between measured and modeled concentrations. Nevertheless, from the calculations concerning hexokinase, it became clear that trehalose 6-phosphate plays an important role in the metabolic part of the regulation (*ρ_m,trehalose_*_ 6-P_ = −1.6).

For the respiratory culture (D = 0.1 h^−1^) it is less straightforward to link the observed metabolic regulation to specific metabolite concentrations. The reason is that the model underestimates the observed increase in flux upon nitrogen starvation. Again the glucose transporter exerts most of the flux control (0.5–0.6 before starvation, 0.9–1.0 after starvation; Table S12 and S13), but since the *V_max_* of the transporter diminished, it tends to decrease rather than increase the glycolytic flux. The *V_max_* of phosphoglucose isomerase, pyruvate kinase and pyruvate decarboxylase increased, but as none of them exerted flux control, this did not lead to a flux increase in the model. In reality there may be a mechanism that confers flux control to pyruvate kinase or pyruvate decarboxylase, which then would explain the increase of the flux. Not knowing what caused the discrepancy of the flux regulation between model and experiment, we are hesitant to speculate on the precise nature of the metabolic regulation.

## Discussion

In this study we have shown that realistic kinetic modeling of biochemical pathways based on independently measured biochemical parameters is possible, provided that parameters are measured under physiological conditions and known allosteric regulation loops are taken into account. Along these lines we have improved the yeast glycolysis model of Teusink *et al.* [12], such that it could be validated for a wider range of experimental conditions.

The key importance of physiological enzyme assays for kinetic modeling of metabolism may seem trivial, but in practice it is not. Until now the effect of *in vivo*-like parameters had never been tested rigorously. Databases are full of parameters measured under a variety of assay conditions, often far from physiological [30]. In order to build metabolic computer models *ab initio*, we will need to redetermine most of the kinetic parameters. It has been doubted if it is possible at all to determine kinetic parameters with sufficient accuracy to simulate the *in vivo* behavior of metabolic pathways quantitatively [13]. A proposed solution is to simplify the kinetic equations and obtain kinetic parameters by fitting them to the experimentally determined fluxes and concentrations [31]. Although this is likely to yield an accurate description of pathway behavior, it will provide little insight into the importance of the biochemical interactions that give rise to such behavior. Moreover, simplified and fitted models will predict pathway behavior accurately for the conditions in which they have been fitted, but they have no predictive power outside the experimental conditions for which they were developed. The latter will require quantitative insight in the non-linearities of the actual kinetics of the enzymes in the network.

The original Teusink *et al.* [12] model failed to reach a steady state if applied to glucose-limited cultures when they receive a high glucose concentration. In such a situation the model - but not the real cell - develops a ‘turbo’ phenotype: the ATP-stimulated synthesis of fructose 1,6-bisphosphate in upper glycolysis persistently exceeds its degradation in lower glycolysis. A negative feedback loop at the beginning of the pathway prevents this ‘turbo’ effect [24]. In yeast glycolysis this role is (at least partially) fulfilled by the inhibition of hexokinase by trehalose 6-phosphate [24]. While allosteric regulation of hexokinase was lacking in the original model of Teusink *et al.* [12], we show that it is required under the experimental conditions of the present study. In particular at D = 0.35 h^−1^, we could demonstrate that it was the main regulator of hexokinase *in vivo*. In addition we show that the feedforward activation of pyruvate kinase by fructose 1,6-bisphosphate is essential to avoid accumulation of metabolites in lower glycolysis, particularly 3-phosphoglycerate, 2-phosphoglycerate and phosphoenolpyruvate (Figure 3, cf. panels K and O). Studying only one steady state, Teusink *et al.* [12] omitted the positive feedforward regulation of pyruvate kinase by fructose 1,6-bisphosphate and thereby had pyruvate kinase maximally active. The reason was that the measured fructose 1,6-bisphosphate concentration was close to saturation of pyruvate kinase and was supposed to have no effect. The positive feedforward regulation of pyruvate kinase, however, does explain the dynamic downregulation of 3-phosphoglycerate, 2-phosphoglycerate and phosphoenolpyruvate after a glucose addition to a glucose limited chemostat, because then fructose 1,6-bisphosphate increases from a low to a high concentration [26], [32]. Even though pyruvate kinase has a low elasticity coefficient towards fructose 1,6-bisphosphate under the steady-state conditions of the present study (elasticity coefficients are in between 0.001 and 0.01), it is apparently sufficient to shift the balance from an accumulation of the metabolites of lower glycolysis towards a stable steady state of that lower glycolysis, corresponding to the experimental data.

The model developed in this study was first applied to four steady-state conditions. In spite of the differences in enzyme and transport capacities, which were the input to the model, the fluxes and metabolite concentrations in the model changed little between the four conditions. However, the doubling of the concentration of fructose 1,6-bisphosphate between D = 0.1 h^−1^ and 0.35 h^−1^ (non-starved) was reproduced correctly by the model (Figure 2 and Table S7and S9). Also the homeostasis of the concentrations of 3-phosphoglycerate, 2-phosphoglycerate and phosphoenolpyruvate between all four conditions was predicted by the model. The above-mentioned activation of pyruvate kinase by fructose 1,6-bisphosphate played an important role herein.

The new model described the dynamic response towards a glucose upshift surprisingly accurately. The only exceptions were the concentrations of glucose 6-phosphate and fructose 6-phosphate, which qualitatively matched the experiment, but the absolute values of which were 5 times lower in the model than in the experiment. We hypothesized that this might be due to the side branches from glucose 6-phosphate towards trehalose and glycogen. In the model these were fixed for lack of dynamic information, but they are likely to change in time. We explored this hypothesis in the model by modulating the branching fluxes and confirmed that the calculated fructose 6-phosphate and glucose 6-phosphate could be adjusted to their experimental values. However, the good fit for the fructose-1,6-bisphosphate concentration was then lost, suggesting a more complex mechanism in which regulation of phosphofructokinase is also involved.

In this study we fixed the ATP, ADP and AMP concentrations, since quantitative kinetic information about ATP utilization was lacking. Instead of variable and mutually dependent ATP, ADP and AMP concentrations, we inserted the measured concentrations as fixed parameters. This choice made the model unsuitable to study phenomena in which ATP dynamics are involved, such as glycolytic oscillations [33]. In the long run this is a limitation. For the purpose of this study, however, to study *in vivo* regulation of the glycolytic enzymes, it was the best option. ATP utilization consists of many processes of which the kinetics are ill-known. Moreover, the sum of ATP, ADP and AMP is not constant during the initial dynamics of glycolysis [34]. Proper modeling of ATP metabolism would have been a study in itself. Future modeling of the dynamics of glycolysis, however, should involve ATP metabolism explicitly. A pragmatic solution to deal with its complexity might be to fit simplified kinetics to the branches of ATP utilization and nucleotide metabolism, thus generating hybrid models in which different levels of detail and explanatory power are combined [35]. The same arguments apply to other side branches.

The *V_max_* values that were used as input to the model were measured at a single pH of 6.8. This was the pH measured in the cytosol of respiratory cells (D = 0.1 h^−1^) [21]. After glucose addition to a glucose-limited chemostat, however, the intracellular pH drops immediately to approximately 5.3, but after 10 seconds the pH increased to pH 5.8 [36]. This may affect the equilibrium constant of the reaction in which protons are involved, *i.e.* glyceraldehyde-3-phosphate dehydrogenase and alcohol dehydrogenase. When the *K_eq_* of alcohol dehydrogenase was changed 10-fold to correct for the pH drop of one unit (from 6.8 to 5.8) the model simulation showed a only a slight difference. In contrast, a 10-fold decrease in the *K_eq_* of glyceraldehyde-3-phosphate dehydrogenase led to accumulation of the intermediates in upper glycolysis. However, besides a change in *K_eq_*, the *V_max_* and the *K_m_* values of the enzymes could also change by a change in pH. The pK*_a_* values of some of the intermediate metabolites, *e.g.* glyceraldehyde 3-phosphate and fructose 6-phosphate, are around 6.5, which implies that a drop in intracellular pH should affect the apparent affinity of the enzymes towards these metabolites. In addition, the isoelectric point of the glycolytic enzymes is in the range of 5–8. Thus a change in pH may affect the protonation of the enzymes, which in principle could alter their *V_max_* as well as their *K_m_* values. We did not observe changes in *V_max_* of glycolytic enzymes in a pH range from 6.5 to 7 (data not shown).

We have to stress that even the improved model of yeast glycolysis does not match the experimental fluxes and metabolite concentrations perfectly. The key progress of this study is that being precise about allosteric regulation and *in vivo*-like assay conditions improves the match between experiment and model substantially, and for a number of different conditions. For an even more stringent test of the paradigm that *in vivo* metabolism is understandable in terms of *in vitro* biochemistry, the obvious next step is to reconsider *all* kinetic parameters, including *K_m_* and *K_i_* values. Apparent *K_m_* and *K_i_* values depend strongly on binding equilibria of metabolites to protons and other cations in the cell. Wu *et al.* took such ion-binding equilibria into account and showed that it improved model predictions of mitochondrial bioenergetics substantially [37], [38]. The protonation state and conformation of the active site of the enzyme in the intracellular environment will, however, be equally important for the affinity of a metabolite for an enzyme. For the short-term we therefore suggest that the redetermination of affinity constants under *in vivo*-like conditions will be unavoidable. The next stage will then be the characterization of pure enzymes and insertion of the properties of isoenzymes into integral models. This requires quantitative proteomics of isoenzymes as well as a strict control of enzyme quality during purification, and has been beyond the scope of this study.

The Silicon Cell philosophy is to construct models of well-defined pathways, which should subsequently be coupled to each other to expand the network [39]. For this approach to be successful, the use of *in vivo*-like enzyme assay conditions will be essential. This outcome may not be surprising, yet we could not have anticipated that it would have been sufficient to get such a good description of glycolytic dynamics. We must be prepared that other pathways may depend on more complex regulation than yeast glycolysis under the conditions of study. In cases where, for instance, enzyme-enzyme complexes, channeling of metabolites or rapid posttranslational modifications determine the pathway dynamics, we will need new means to analyze the *in vivo* biochemistry quantitatively. This will be the next challenge for molecular systems biology.

## Materials and Methods

### Growth and nitrogen-starvation conditions

The haploid, prototrophic *Saccharomyces cerevisiae* strain CEN.PK113-7D (*MATa*, *MAL2-8^c^*, *SUC2*) was cultivated in an aerobic, glucose-limited chemostat (1 l laboratory fermentor, Applikon) as described in detail by Van Hoek *et al.* [40]. Chemostat cultures were fed with defined mineral medium [41] in which glucose (42 mM) was the growth-limiting nutrient. Yeast cells were grown under either respiratory or respirofermentative conditions at dilution rates of 0.1 and 0.35 h^−1^, respectively.

For the nitrogen-starvation experiments the same defined mineral medium was used as for the chemostat culture, except that it lacked ammonium sulfate and contained an excess of glucose. The addition of glucose served to prevent additional starvation for the carbon source. Yeast cells harvested from steady-state chemostats were washed with ice-cold starvation medium and resuspended in starvation medium. Cells were brought back in a new fermentor in batch mode (start volume was around 1 litre) at otherwise the same conditions as during chemostat cultivation (for a detailed description see [23]).

### Glucose-transport assay

Zero-trans influx of ^14^C-labeled glucose was measured in a 5-s uptake assay as described by Walsh *et al.* [42] with the modifications of Rossell *et al.* [43] at 30°C. The range of glucose concentrations was between 0.25 and 225 mM. Irreversible Michaelis-Menten equations without product inhibition were fitted to the data by nonlinear regression.

### V_max_ measurements under in vivo-like assay conditions

Cell-free extracts were prepared freshly by the FastPrep® method described in Van Eunen *et al.* [21]. *V_max_* assays were carried out with the prepared extracts via NAD(P)H-linked assays, at 30°C in a Novostar spectrophotometer (BMG Labtech) as described in detail in [21].

The standardized *in vivo*-like assay medium [21] contained 300 mM potassium, 245 mM glutamate, 50 mM phosphate, 20 mM sodium, 2 mM free magnesium, 5–10 mM sulphate, and 0.5 mM calcium. For the addition of magnesium, it was taken into account that ATP, ADP, NADP and TPP bind magnesium with a high affinity. The amount of magnesium added equaled the summed concentration of these coenzymes plus 2 mM, such that the free magnesium concentration was in slight excess of 2 mM. Since the sulfate salt of magnesium was used, the sulfate concentration in the final assay medium varied between 2.5 and 10 mM. The concentrations of the substrates and coupling enzymes for each individual enzyme assay are given in the supporting information Text S1.

### Model description

The glycolytic model of Teusink *et al.* [12] was the starting point for this study. The model as it was used here, is depicted in Figure 1. Starting from the original Teusink *et al.* model [12] the following modifications were made, based on new insights and in order to tailor the model to the experimental conditions of this study:

1. The *V_max_* values of all glycolytic and fermentative enzymes and the *V_max_* and affinity constant of glucose transport were measured in extracts of cells grown under the growth and starvation conditions of this study. In Van Eunen *et al.* [23] they had been measured under assay conditions optimized for each enzyme; here we report on *in vivo*-like assays (see above) for the same biological samples (Table S4). For most of the remaining kinetic parameters we have used the values of Teusink *et al.* [12]. The only exceptions were the *K_m_* values of glyceraldehyde-3-phosphate dehydrogenase. *In vivo*-like kinetic data of glyceraldehyde-3-phosphate dehydrogenase used in the model is given in Table S4. If not mentioned otherwise the glyceraldehyde-3-phosphate dehydrogenase parameters and the *V_max_* values measured under *in vivo*-like assay conditions were used.
2. In the original Teusink *et al.* model the branching fluxes to trehalose and glycogen were fixed at their measured values. Under the conditions described here, the glycogen flux was negligible (data not shown) and therefore not included. The trehalose, glycerol and succinate fluxes were fixed at the values measured in our study (Table 1). To prevent a redox imbalance in the model we did not fix the flux to acetate. Instead it was made proportional to the acetaldehyde concentration with a rate constant of 0.5 min^−1^.
3. In the original model [12] the net ATP produced by glycolysis was consumed in a lumped reaction of ATP utilization. This resulted in variable and mutually dependent ATP, ADP and AMP concentrations. Since information about the kinetics of ATP utilization was lacking and moreover not the focus of this study, we decided to remove the ATP utilization from the model and instead inserted the measured concentrations of ATP, ADP and AMP as fixed parameters.
4. The known inhibition of hexokinase by trehalose 6-phosphate had not been included in the original model [12]. Yet, it is thought to play an important role in the regulation of glycolysis, particularly to prevent an imbalance between the upper and lower part of the pathway [24]. Trehalose 6-phosphate is an inhibitor of hexokinase that competes with its substrate glucose. Different *K_i_* values for the different hexokinases of yeast have been reported. Glucokinase was not inhibited by trehalose 6-phosphate, while the *K_i_* values of hexokinase 1 and 2 were 0.2 mM and 0.04 mM, respectively [44]. Since the distribution of the isoenzymes is not known for the experimental conditions studied here, both *K_i_* values were used as indicated in the text. The kinetic equation of hexokinase was modified to the following:
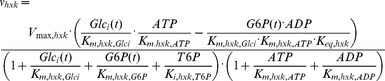
(1)
5. Finally, the *K_m_* of pyruvate decarboxylase in the original model (4.3 mM) [12] had been obtained from Boiteux and Hess [45] based on an intracellular phosphate concentration of 25 mM. However, we have measured the pyruvate decarboxylase activity at a phosphate concentration of 50 mM, which is likely to be the intracellular concentration under the growth conditions studied here [29]. Based on the data of Boiteux and Hess [45] we calculated a *K_m_* value of pyruvate decarboxylase for pyruvate of 6.36 mM at 50 mM phosphate and the new value was inserted in the model.
6. The known activation of pyruvate kinase by fructose 1,6-bisphosphate had not been included in the original model [12]. However, it might play an important role, especially in cases where fructose 1,6-bisphospohate is below the concentration for which the activation of pyruvate kinase is at a maximum. We have implemented this allosteric regulation of pyruvate kinase according to the rate equation of Rizzi *et al.* [11], in which the activation by fructose 1,6-bisphosphate depended on the ATP concentration.
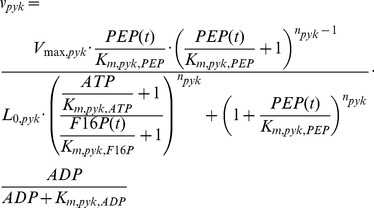
(2)All experimental data were converted to intracellular units (mM·min^−1^ for rates and mM for concentrations) by assuming a yeast cytosolic volume of 3.75 µl.mg cell protein^−1^ [46]. The final model including all equations and the parameters that were constant for the four conditions studied is given in the supporting information material Text S2, S3 and Table S1, S2, S3, S4, S5, S6.

## Supporting Information

Table S1 **Fixed parameters under all four conditions.**
(PDF)

Table S2 **Concentrations of metabolites fixed under all four conditions.**
(PDF)

Table S3 **Initial metabolite concentrations in mM used in the glycolytic model.**
(PDF)

Table S4 **Kinetic parameters measured under** ***in vivo*** **-like conditions and implemented in the glycolytic model under the four conditions studied (new measurements).**
(PDF)

Table S5 ***V_max_*** **values measured under ‘** ***V_max_*** **optimized assay’ conditions.**
(PDF)

Table S6 **Parameter used to simulate the upshift of the extracellular glucose concentration.**
(PDF)

Table S7 **Predictions from the model of yeast glycolysis for the non-starved cells from the respiratory culture (D = 0.1 h^−1^), compared to experimental data from** [**23**] **.**
(PDF)

Table S8 **Predictions from the model of yeast glycolysis for the 4 h N-starved cells from the respiratory culture (D = 0.1 h^−1^), compared to experimental data from** [**23**] **.**
(PDF)

Table S9 **Predictions from the model of yeast glycolysis for the non-starved cells from the respirofermentative culture (D = 0.35 h^−1^), compared to experimental data from** [**23**] **.**
(PDF)

Table S10 **Predictions from the model of yeast glycolysis for the 4 h N-starved cells from the respirofermentative culture (D = 0.35 h^−1^), compared to experimental data from** [**23**] **.**
(PDF)

Table S11 **Response coefficients of the concentration of F16BP towards the model parameters** ***p*** **.**
(PDF)

Table S12 **Flux Control Coefficients, D = 0.1 h^−1^, Non-starved.**
(PDF)

Table S13 **Flux Control Coefficients, D = 0.1 h^−1^, 4 h nitrogen-starved.**
(PDF)

Table S14 **Flux Control Coefficients, D = 0.35 h^−1^, non-starved.**
(PDF)

Table S15 **Flux Control Coefficients, D = 0.35 h^−1^, 4 h nitrogen-starved.**
(PDF)

Table S16 **Comparison of the regulation coefficients presented in** [**23**] **(** ***V_max_*** **values measured under enzyme-optimized conditions) and those re-calculated with the** ***V_max_*** **values measured under** ***in vivo*** **-like conditions, but using the same changes in flux.**
(PDF)

Text S1 **Concentrations of substrates and coupling enzymes in the** ***in vivo*** **-like assays for each individual enzyme.**
(PDF)

Text S2 **Ordinary differential equations.**
(PDF)

Text S3 **Rate equations.**
(PDF)
