# Supplementary material for: Testing Biochemistry Revisited: How In Vivo Metabolism Can Be Understood from In Vitro Enzyme Kinetics
Source: PLoS Comput Biol. 2012 Apr 26;8(4):e1002483. doi: 10.1371/journal.pcbi.1002483 (PMC3343101; doi:10.1371/journal.pcbi.1002483)
Supplement: Table S1 — Fixed parameters under all four conditions. (PDF) [file pcbi.1002483.s001.pdf]

Table S1 Fixed parameters under all four conditions. Values were taken from [1].

Parameter	Value		Parameter	Value	
$K_{eq,glt}$	1		$K_{m,pgk,BPG}$	0.003	mM
P	0.91		$K_{m,pgk,P3G}$	0.53	mM
$K_{m,hk,GLCi}$	0.08	mM	$K_{m,pgk,ADP}$	0.2	mM
$K_{m,hk,G6P}$	30	mM	$K_{m,pgk,ATP}$	0.3	mM
$K_{m,hk,ATP}$	0.15	mM	$K_{eq,pgk}$	3200	
$K_{m,hk,ADP}$	0.23	mM	$K_{m,gpm,P3G}$	1.2	mM
$K_{i,hk,T6P}$	0.2/0.04	mM	$K_{m,gpm,P2G}$	0.08	mM
$K_{eq,hk}$	3800		$K_{eq,gpm}$	0.19	
$K_{m,pgi,G6P}$	1.4	mM	$K_{m,eno,P2G}$	0.04	mM
$K_{m,pgi,F6P}$	0.3	mM	$K_{m,eno,PEP}$	0.5	mM
$K_{eq,pgi}$	0.314		$K_{eq,eno}$	6.7	
gR_{PFK}	5.12		$K_{m,pyk,PEP}$	0.19	mM
$L_{0,PFK}$	0.66		$K_{m,pyk,ADP}$	0.3	mM
$K_{m,pfk,F6P}$	0.1	mM	$K_{m,pyk,ATP}$	9.3	mM
$K_{m,pfk,ATP}$	0.71	mM	n_{pyk}	4	
$C_{pfk,ATP}$	3		$L_{0,pyk}$	60000	
$C_{i,pfk,ATP}$	100		$K_{m,pyk,F16P}$	0.2	mM
$C_{i,pfk,AMP}$	0.0845		$K_{m,pdc,PYR}$	6.36	mM
$C_{i,pfk,F16BP}$	0.397		NH_{PDC}	1.9	
$C_{i,pfk,F26BP}$	0.0174		$K_{m,adh,ACALD}$	1.11	mM
$K_{pfk,ATP}$	0.65	mM	$K_{m,adh,ETOH}$	17	mM
$K_{pfk,AMP}$	0.0995	mM	$K_{m,adh,NADH}$	0.11	mM
$K_{pfk,F16BP}$	0.111	mM	$K_{m,adh,NAD}$	0.17	mM
$K_{pfk,F26BP}$	0.000682	mM	$K_{i,adh,ACALD}$	1.1	mM
$K_{m,ald,F16BP}$	0.3	mM	$K_{i,adh,ETOH}$	90	mM
$K_{m,ald,GAP}$	2	mM	$K_{i,adh,NADH}$	0.031	mM
$K_{m,ald,DHAP}$	2.4	mM	$K_{i,adh,NAD}$	0.92	mM
$K_{m,ald,GAPi}$	10	mM	$K_{eq,adh}$	6.9×10^{-5}	
$K_{eq,ald}$	0.069		K_{ace}	0.5	
$K_{eq,tpi}$	0.045				

References

1. Teusink B, Passarge J, Reijenga CA, Esgalhado E, van der Weijden CC, et al. (2000) Can yeast glycolysis be understood in terms of in vitro kinetics of the constituent enzymes? Testing biochemistry. Eur J Biochem 267: 5313-5329.
