# Supplementary material for: Testing Biochemistry Revisited: How In Vivo Metabolism Can Be Understood from In Vitro Enzyme Kinetics
Source: PLoS Comput Biol. 2012 Apr 26;8(4):e1002483. doi: 10.1371/journal.pcbi.1002483 (PMC3343101; doi:10.1371/journal.pcbi.1002483)
Supplement: Table S2 — Concentrations of metabolites fixed under all four conditions. (PDF) [file pcbi.1002483.s002.pdf]

Table S2 Concentrations of metabolites fixed under all four conditions.

Metabolite	Concentration (mM)
GLC_o	50
ETOH	25
NADt	1.59
