# Supplementary material for: Testing Biochemistry Revisited: How In Vivo Metabolism Can Be Understood from In Vitro Enzyme Kinetics
Source: PLoS Comput Biol. 2012 Apr 26;8(4):e1002483. doi: 10.1371/journal.pcbi.1002483 (PMC3343101; doi:10.1371/journal.pcbi.1002483)
Supplement: Table S3 — Initial metabolite concentrations in mM used in the glycolytic model. (PDF) [file pcbi.1002483.s003.pdf]

Table S3 Initial metabolite concentrations in mM used in the glycolytic model. These concentrations were measured in the corresponding steady states.

Metabolite	D = 0.1 h^{-1} Non-starved	D = 0.1 h^{-1} 4h N-starved	D = 0.35 h^{-1} Non-starved	D = 0.1 h^{-1} 4h N-starved
$GLCi_0$ *	0.10	0.10	0.10	0.10
$G6P_0$	3.80	4.22	5.38	4.45
$F6P_0$	0.74	0.80	1.01	0.78
$F16BP_0$	11.80	14.63	27.03	16.39
$TRIO_0$ *	1.00	1.00	1.00	1.00
BPG_0 *	1.00×10^{-5}	1.00×10^{-5}	1.00×10^{-5}	1.00×10^{-5}
$P3G_0$	0.69	0.96	1.09	1.00
$P2G_0$	0.09	0.13	0.15	0.13
PEP_0	0.10	0.12	0.11	0.12
PYR_0	2.76	3.48	5.32	3.90
$ACALD_0$ *	0.04	0.04	0.04	0.04
$NADH_0$ *	0.29	0.29	0.29	0.29

*These metabolites were not measured and thus we used the initial concentrations in the model of Teusink *et al.* [1]

References

1. Teusink B, Passarge J, Reijenga CA, Esgalhado E, van der Weijden CC, et al. (2000) Can yeast glycolysis be understood in terms of in vitro kinetics of the constituent enzymes? Testing biochemistry. Eur J Biochem 267: 5313-5329.
