# Supplementary material for: Testing Biochemistry Revisited: How In Vivo Metabolism Can Be Understood from In Vitro Enzyme Kinetics
Source: PLoS Comput Biol. 2012 Apr 26;8(4):e1002483. doi: 10.1371/journal.pcbi.1002483 (PMC3343101; doi:10.1371/journal.pcbi.1002483)
Supplement: Table S4 — Kinetic parameters measured under in vivo -like conditions and implemented in the glycolytic model under the four conditions studied (new measurements). (PDF) [file pcbi.1002483.s004.pdf]

Table S4 Kinetic parameters measured under *in vivo*-like conditions and implemented in the glycolytic model under the four conditions studied (new measurements).

Parameter	D = 0.1 h^{-1} Non-starved	D = 0.1 h^{-1} 4h N-starved	D = 0.35 h^{-1} Non-starved	D = 0.35 h^{-1} 4h N-starved	
$V_{max,glt}$	220	121	201	95	$mM.min^{-1}$
$K_{m,glt,GLC}$	1.6	11.0	0.9	7.0	mM
$V_{max,hk}$	285	223	258	227	$mM.min^{-1}$
$V_{max,pgi}$	808	852	903	856	$mM.min^{-1}$
$V_{max,pfk}$	213	165	179	93	$mM.min^{-1}$
$V_{max,ald}$	189	153	200	161	$mM.min^{-1}$
$V^{+}_{max,gapdh}$	1859 (2090)[a]	1075 (1514)[a]	1496	853 (1450)[a]	$mM.min^{-1}$
$V^{-}_{max,gapdh}$	1211	877	867	840	$mM.min^{-1}$
$K_{m,gapdh,GAP}$	2.48	1.15	0.39	1.41	mM; taken from [1]
$K_{m,gapdh,NAD}$	2.92	2.95	2.85	2.62	mM
$K_{m,gapdh,NADH}$	0.022	0.10	0.007	0.014	mM; taken from [1]
$K_{m,gapdh,BPG}$	1.18[b]	0.15[b]	0.51[b]	1.43[b]	mM; calculated
$K_{eq,gapdh}$	0.0056	0.0056	0.0056	0.0056	Dimensionless; taken from [1]
$V^{-}_{max,pgk}$	2670	3030	2416	1962	$mM.min^{-1}$
$V_{max,gpm}$	856	748	871	403	$mM.min^{-1}$
$V_{max,eno}$	357	285	485	272	$mM.min^{-1}$
$V_{max,pyk}$	559	636	677	480	$mM.min^{-1}$
$V_{max,pdc}$	248	297	335	172	$mM.min^{-1}$
$V^{-}_{max,adh}$	817	744	856	744	$mM.min^{-1}$

[a] The value between brackets is the V_{max} value calculated using the Haldane relationship, the K_{eq}, the reverse V_{max} of the specific condition and the K_m values of the non-starved cells from the respirofermentative culture (D = 0.35 h^{-1})

[b] The K_m for BPG was calculated using the Haldane relationship, the measured V_{max} values, the K_m values for the other substrates and products, and the K_{eq}.

References

1. Teusink B, Passarge J, Reijenga CA, Esgalhado E, van der Weijden CC, et al. (2000) Can yeast glycolysis be understood in terms of in vitro kinetics of the constituent enzymes? Testing biochemistry. Eur J Biochem 267: 5313-5329.
