# Supplementary material for: Testing Biochemistry Revisited: How In Vivo Metabolism Can Be Understood from In Vitro Enzyme Kinetics
Source: PLoS Comput Biol. 2012 Apr 26;8(4):e1002483. doi: 10.1371/journal.pcbi.1002483 (PMC3343101; doi:10.1371/journal.pcbi.1002483)
Supplement: Table S5 — Vmax values measured under ‘ Vmax optimized assay’ conditions. (PDF) [file pcbi.1002483.s005.pdf]

Table S5 V_{max} values measured under 'V_{max} optimized assay' conditions (data were taken from [1]).

Parameter	D = 0.1 h^{-1} Non-starved	D = 0.1 h^{-1} 4h N-starved	D = 0.35 h^{-1} Non-starved	D = 0.35 h^{-1} 4h N-starved	
$V_{max,hk}$	483	326	552	498	$mM.min^{-1}$
$V_{max,pgi}$	1665	1573	1141	1117	$mM.min^{-1}$
$V_{max,pfk}$	173	133	98	74	$mM.min^{-1}$
$V_{max,ald}$	165	122	251	147	$mM.min^{-1}$
$V^{+}_{max,gapdh}$	296[a]	241[a]	198[a]	205[a]	$mM.min^{-1}$
$V^{-}_{max,gapdh}$	1646	1341	1101	1141	$mM.min^{-1}$
$V^{-}_{max,pgk}$	2808	3063	1662	1364	$mM.min^{-1}$
$V_{max,gpm}$	1493	n.d.	1503	543	$mM.min^{-1}$
$V_{max,eno}$	264	n.d.	285	n.d.	$mM.min^{-1}$
$V_{max,pyk}$	779	761	965	773	$mM.min^{-1}$
$V_{max,pdc}$	153	224	219	72	$mM.min^{-1}$
$V^{-}_{max,adh}$	2696	2025	438	289	$mM.min^{-1}$

[a] Forward V_{max} value of GAPDH was not measured but calculated with the Haldane relationship using the kinetic parameters from [2].

n.d.: Not determined due to practical issues.

References

1. van Eunen K, Dool P, Canelas AB, Kiewiet J, Bouwman J, et al. (2010) Time-dependent regulation of yeast glycolysis upon nitrogen starvation depends on cell history. IET Syst Biol 4: 157-168.
2. Teusink B, Passarge J, Reijenga CA, Esgalhado E, van der Weijden CC, et al. (2000) Can yeast glycolysis be understood in terms of in vitro kinetics of the constituent enzymes? Testing biochemistry. Eur J Biochem 267: 5313-5329.
