# Supplementary material for: Testing Biochemistry Revisited: How In Vivo Metabolism Can Be Understood from In Vitro Enzyme Kinetics
Source: PLoS Comput Biol. 2012 Apr 26;8(4):e1002483. doi: 10.1371/journal.pcbi.1002483 (PMC3343101; doi:10.1371/journal.pcbi.1002483)
Supplement: Table S6 — Parameter used to simulate the upshift of the extracellular glucose concentration. (PDF) [file pcbi.1002483.s006.pdf]

Table S6 Parameter used to simulate the upshift of the extracellular glucose concentration.

Parameter	Aerobic glucose-limited chemostat (D = 0.1 h^{-1})	
Enzyme kinetics		
$V_{max,glt}$	160	mM.min^{-1}
$K_{m,glt,GLC}$	1	mM
$V_{max,hk}$	213	mM.min^{-1}
$K_{i,hk,T6P}$	0.04	mM
$V_{max,pgi}$	787	mM.min^{-1}
$V_{max,pfk}$	213	mM.min^{-1}
$V_{max,ald}$	310	mM.min^{-1}
$V^{+}_{max,gapdh}$	1300	mM.min^{-1}
$V^{-}_{max,gapdh}$	853	mM.min^{-1}
$K_{m,gapdh,GAP}$	0.21	mM;<br>Taken from [1]
$K_{m,gapdh,NAD}$	2.8	mM
$K_{m,gapdh,NADH}$	0.06	mM;<br>Taken from [1]
$K_{m,gapdh,BPG}$	0.036	mM
$K_{eq,gapdh}$	0.0056	Taken from [1]
$V^{-}_{max,pgk}$	2512	mM.min^{-1}
$V_{max,gpm}$	856	mM.min^{-1}
$V_{max,eno}$	357	mM.min^{-1}
$V_{max,pyk}$	820	mM.min^{-1}
$V_{max,pdc}$	395	mM.min^{-1}
$V^{-}_{max,adh}$	932	mM.min^{-1}
Regulators		
ATP	3	mM
ADP	1	mM
AMP	0.3	mM
F26BP	0.014	mM
T6P	0.2	mM

References

1. Teusink B, Passarge J, Reijenga CA, Esgalhado E, van der Weijden CC, et al. (2000) Can yeast glycolysis be understood in terms of in vitro kinetics of the constituent enzymes? Testing biochemistry. Eur J Biochem 267: 5313-5329.
