# Supplementary material for: Testing Biochemistry Revisited: How In Vivo Metabolism Can Be Understood from In Vitro Enzyme Kinetics
Source: PLoS Comput Biol. 2012 Apr 26;8(4):e1002483. doi: 10.1371/journal.pcbi.1002483 (PMC3343101; doi:10.1371/journal.pcbi.1002483)
Supplement: Table S7 — Predictions from the model of yeast glycolysis for the non-starved cells from the respiratory culture (D = 0.1 h−1), compared to experimental data from [23] . (PDF) [file pcbi.1002483.s007.pdf]

Table S7 Predictions from the model of yeast glycolysis for the non-starved cells from the respiratory culture (D = 0.1 h^{-1}), compared to experimental data from [1]. Bold numbers are used for Fig. 2.

	**Experiment**	**Model**			
		GAPDH parameters of D = 0.1 h^{-1}, non starved		GAPDH parameters of D = 0.35 h^{-1}, non starved	
		$K_{i,hk,T6P}$ 0.2 mM	$K_{i,hk,T6P}$ 0.04 mM	$K_{i,hk,T6P}$ 0.2 mM	$K_{i,hk,T6P}$ 0.04 mM
Flux					
HXT-HXK	94 ± 4	127	84	127	**84**
PGI-ALD	82 ± 3	131	88	131	**88**
GAPDH-ADH	147 ± 4	245	158	245	**158**
Metabolites					
G6P	3.8 ± 0.1	3.9	2.0	3.8	**1.8**
F6P	0.74 ± 0.01	0.77	0.45	0.76	**0.39**
F16BP	12 ± 1	5801	495	122	**12**
3PG+2PG	0.78 ± 0.02	4.1	1.0	4.1	**1.0**
PEP	0.10 ± 0.01	0.25	0.11	0.25	**0.11**
PYR	2.8 ± 0.6	53	8.4	53	**8.4**

References

1. van Eunen K, Dool P, Canelas AB, Kiewiet J, Bouwman J, et al. (2010) Time-dependent regulation of yeast glycolysis upon nitrogen starvation depends on cell history. IET Syst Biol 4: 157-168.
