# Supplementary material for: Testing Biochemistry Revisited: How In Vivo Metabolism Can Be Understood from In Vitro Enzyme Kinetics
Source: PLoS Comput Biol. 2012 Apr 26;8(4):e1002483. doi: 10.1371/journal.pcbi.1002483 (PMC3343101; doi:10.1371/journal.pcbi.1002483)
Supplement: Table S8 — Predictions from the model of yeast glycolysis for the 4 h N-starved cells from the respiratory culture (D = 0.1 h−1), compared to experimental data from [23] . (PDF) [file pcbi.1002483.s008.pdf]

Table S8 Predictions from the model of yeast glycolysis for the 4h N-starved cells from the respiratory culture (D = 0.1 h^{-1}), compared to experimental data from [1]. Bold numbers are used for Fig. 2.

	Experiment	Model			
		GAPDH parameters of D = 0.1 h^{-1}, 4h N-starved		GAPDH parameters of D = 0.35 h^{-1}, non starved	
		$K_{i,hk,T6P}$ 0.2 mM	$K_{i,hk,T6P}$ 0.04 mM	$K_{i,hk,T6P}$ 0.2 mM	$K_{i,hk,T6P}$ 0.04 mM
Flux					
HXT-HXK	139 ± 10	97	91	97	**91**
PGI-ALD	119 ± 5	101	96	101	**96**
GAPDH-ADH	213 ± 11	177	166	177	**166**
Metabolites					
G6P	4.2 ± 0.1	3.7	3.3	3.6	**3.2**
F6P	0.80 ± 0.04	0.82	0.75	0.81	**0.73**
F16BP	15 ± 0	1412	949	78	**55**
3PG+2PG	1.1 ± 0.0	2.0	1.6	2.0	**1.6**
PEP	0.12 ± 0.00	0.12	0.11	0.12	**0.11**
PYR	3.5 ± 0.3	7.8	7.2	7.8	**7.2**

References

1. van Eunen K, Dool P, Canelas AB, Kiewiet J, Bouwman J, et al. (2010) Time-dependent regulation of yeast glycolysis upon nitrogen starvation depends on cell history. IET Syst Biol 4: 157-168.
