# Supplementary material for: Testing Biochemistry Revisited: How In Vivo Metabolism Can Be Understood from In Vitro Enzyme Kinetics
Source: PLoS Comput Biol. 2012 Apr 26;8(4):e1002483. doi: 10.1371/journal.pcbi.1002483 (PMC3343101; doi:10.1371/journal.pcbi.1002483)
Supplement: Table S9 — Predictions from the model of yeast glycolysis for the non-starved cells from the respirofermentative culture (D = 0.35 h−1), compared to experimental data from [23] . (PDF) [file pcbi.1002483.s009.pdf]

Table S9 Predictions from the model of yeast glycolysis for the non-starved cells from the respirofermentative culture (D = 0.35 h^{-1}), compared to experimental data from [1]. Bold numbers are used for Fig. 2.

	**Experiment**	**Model**	
		GAPDH parameters of D = 0.35 h^{-1}, non starved	
		$K_{i,hk,T6P}$ 0.2 mM	$K_{i,hk,T6P}$ 0.04 mM
Flux			
HXT-HXK	120 ± 6	**90**	54
PGI-ALD	99 ± 6	**88**	52
GAPDH-ADH	177 ± 11	**155**	82
Metabolites			
G6P	5.4 ± 0.2	**1.9**	0.98
F6P	1.0 ± 0.0	**0.4**	0.24
F16BP	27 ± 2	**27**	3.0
3PG+2PG	1.2 ± 0.1	**0.7**	0.32
PEP	0.11 ± 0.01	**0.07**	0.08
PYR	5.3 ± 0.0	**5.8**	3.5

References

1. van Eunen K, Dool P, Canelas AB, Kiewiet J, Bouwman J, et al. (2010) Time-dependent regulation of yeast glycolysis upon nitrogen starvation depends on cell history. IET Syst Biol 4: 157-168.
