# Supplementary material for: Testing Biochemistry Revisited: How In Vivo Metabolism Can Be Understood from In Vitro Enzyme Kinetics
Source: PLoS Comput Biol. 2012 Apr 26;8(4):e1002483. doi: 10.1371/journal.pcbi.1002483 (PMC3343101; doi:10.1371/journal.pcbi.1002483)
Supplement: Table S10 — Predictions from the model of yeast glycolysis for the 4 h N-starved cells from the respirofermentative culture (D = 0.35 h−1), compared to experimental data from [23] . (PDF) [file pcbi.1002483.s010.pdf]

Table S10 Predictions from the model of yeast glycolysis for the 4h N-starved cells from the respirofermentative culture (D = 0.35 h^{-1}), compared to experimental data from [1]. Bold numbers are used for Fig. 2.

	**Experiment**	**Model**			
		GAPDH parameters of D = 0.35 h^{-1}, 4h N-starved		GAPDH parameters of D = 0.35 h^{-1}, non starved	
		$K_{i,hk,T6P}$ 0.2 mM	$K_{i,hk,T6P}$ 0.04 mM	$K_{i,hk,T6P}$ 0.2 mM	$K_{i,hk,T6P}$ 0.04 mM
Flux					
HXT-HXK	83 ± 4	74	73	74	**73**
PGI-ALD	85 ± 4	78	77	78	**77**
GAPDH-ADH	148 ± 9	135	133	134	**133**
Metabolites					
G6P	4.4 ± 0.3	314	18	314	**18**
F6P	0.78 ± 0.09	79	4.6	79	**4.4**
F16BP	16 ± 1	998	948	21	**20**
3PG+2PG	1.1 ± 0.0	1.9	1.8	1.9	**1.8**
PEP	0.12 ± 0.00	0.11	0.10	0.11	**0.10**
PYR	3.9 ± 0.3	12	12	12	**12**

References

1. van Eunen K, Dool P, Canelas AB, Kiewiet J, Bouwman J, et al. (2010) Time-dependent regulation of yeast glycolysis upon nitrogen starvation depends on cell history. IET Syst Biol 4: 157-168.
