# Supplementary material for: Testing Biochemistry Revisited: How In Vivo Metabolism Can Be Understood from In Vitro Enzyme Kinetics
Source: PLoS Comput Biol. 2012 Apr 26;8(4):e1002483. doi: 10.1371/journal.pcbi.1002483 (PMC3343101; doi:10.1371/journal.pcbi.1002483)
Supplement: Table S11 — Response coefficients of the concentration of F16BP towards the model parameters p . (PDF) [file pcbi.1002483.s011.pdf]

Table S11 Response coefficients of the concentration of F16BP towards the model parameters *p*. The response coefficient $R^{j}_{p_i} = \frac{d\ln[F16BP]}{d\ln p_i} = \frac{d[F16BP]}{dp_i}\cdot\frac{p_i}{[F16BP]} \approx \frac{[F16BP]_{+h}-[F16BP]_{-h}}{2\cdot h}\cdot\frac{p_{i,0}}{[F16BP]_0}$ was approximated by either increasing or decreasing the parameter of interest by *h*, which was 1%. This was done for the model versions corresponding to figure 2 in the main text.

Parameter	D = 0.1 h^{-1} Non-starved	D = 0.1 h^{-1} 4h N-starved	D = 0.35 h^{-1} Non-starved	D = 0.1 h^{-1} 4h N-starved
$V_{max,glt}$	2.16	5.25	2.54	3.01
$K_{m,glt,GLCo}$	-0.07	-0.95	-0.05	-0.40
$K_{m,glt,GLCi}$	1.27	0.34	1.36	0.33
$K_{eq,glt}$	0.11	0.09	0.06	0.06
$V_{max,hk}$	2.15	0.86	2.48	0.65
$K_{m,hk,GLCi}$	-1.39	-0.43	-1.42	-0.39
$K_{m,hk,G6P}$	0.00	0.00	0.00	0.01
$K_{i,hk,T6P}$	1.36	0.38	1.33	0.35
$K_{m,hk,ATP}$	-0.30	-0.13	-0.47	-0.10
$K_{m,hk,ADP}$	0.24	0.10	0.40	0.08
$K_{eq,hk}$	0.00	0.00	0.00	0.00
$V_{max,pgi}$	0.00	0.00	0.00	0.00
$K_{m,pgi,G6P}$	0.00	0.00	0.00	0.00
$K_{m,pgi,F6P}$	0.00	0.00	0.00	0.00
$K_{eq,pgi}$	0.00	0.00	0.00	0.01
K_{tre1}	0.11	0.25	0.00	0.17
K_{tre2}	0.00	0.00	-0.11	0.00
$V_{max,pfk}$	0.00	0.01	0.01	1.21
gR_{pfk}	0.00	0.00	0.00	0.17
$L_{0,pfk}$	0.00	0.00	0.00	-0.01
$K_{m,pfk,F6P}$	0.00	0.00	0.00	-0.01
$K_{m,pfk,ATP}$	0.00	0.00	0.00	0.00
$C_{pfk,ATP}$	0.00	0.00	0.00	-0.01
$K_{pfk,AMP}$	0.00	0.00	0.00	-0.01
$C_{pfk,AMP}$	0.00	0.00	0.00	0.00
$K_{i,pfk,ATP}$	0.00	0.00	0.00	0.00
$C_{i,pfk,ATP}$	0.00	0.00	0.00	-0.01
$K_{pfk,F26BP}$	0.00	0.00	0.00	0.00
$C_{pfk,F26BP}$	0.00	0.00	0.00	0.00
$K_{pfk,F16BP}$	0.00	0.00	0.00	0.00
$C_{pfk,F16BP}$	0.00	0.00	0.00	-0.01
$K_{eq,tpi}$	-0.98	-0.99	-0.99	-0.98
$V_{max,ald}$	-0.94	-1.82	-0.85	-0.99
$K_{m,ald,F16P}$	0.05	0.03	0.03	0.04
$K_{m,ald,GAP}$	0.00	0.00	0.00	0.00
$K_{m,ald,DHAP}$	-0.03	-0.02	-0.02	-0.02
$K_{m,ald,GAPi}$	-0.01	-0.04	-0.02	-0.02
$K_{eq,ald}$	-0.95	-0.97	-0.97	-0.96
K_{gly}	-0.46	-0.68	-0.60	-0.63
$V^{+}_{max,gapdh}$	-2.62	-3.24	-3.04	-2.83
$V^{-}_{max,gapdh}$	0.01	0.01	0.00	0.01
$K_{m,gapdh,GAP}$	1.94	2.00	1.98	1.96
$K_{m,gapdh,BPG}$	-0.02	-0.02	-0.01	-0.02
$K_{m,gapdh,NAD}$	1.86	2.31	2.14	1.99
$K_{m,gapdh,NADH}$	-0.50	-0.64	-0.53	-0.48
C_{gapdh}	-2.61	-3.23	-3.04	-2.82
$NADt$	-1.50	-1.85	-1.76	-1.64
$V_{max,pgk}$	0.00	0.00	0.00	0.00

$K_{m,pgk,BPG}$	0.00	0.00	0.00	0.00
$K_{m,pgk,P3G}$	0.00	0.00	0.00	0.00
$K_{m,pgk,ADP}$	0.00	0.00	0.00	0.00
$K_{m,pgk,ATP}$	0.00	0.00	0.00	0.00
$K_{eq,pgk}$	-0.02	-0.02	-0.01	-0.02
$V_{max,gpm}$	-0.01	-0.02	0.00	-0.02
$K_{m,gpm,P3G}$	0.01	0.01	0.00	0.01
$K_{m,gpm,P2G}$	0.00	-0.01	0.00	-0.01
$K_{eq,gpm}$	-0.01	-0.01	0.00	-0.01
$V_{max,eno}$	-0.01	-0.03	0.00	-0.02
$K_{m,eno,P2G}$	0.01	0.01	0.00	0.01
$K_{m,eno,PEP}$	0.00	0.00	0.00	0.00
$K_{eq,eno}$	0.00	-0.01	0.00	-0.01
$V_{max,pyk}$	-0.01	-0.01	0.00	-0.01
$K_{m,pyk,PEP}$	0.01	0.01	0.00	0.01
$K_{m,pyk,ADP}$	0.00	0.00	0.00	0.00
$K_{m,pyk,ATP}$	0.00	0.00	0.00	0.00
$n_{10,pyk}$	0.00	0.00	0.00	0.00
$L_{10,pyk}$	0.00	0.00	0.00	0.00
$K_{m,pyk,F16P}$	0.00	0.00	0.00	0.00
$V_{max,pdc}$	0.00	0.00	0.00	0.00
$K_{m,pdc,PYR}$	0.00	0.00	0.00	0.00
NH_{pdc}	0.00	0.00	0.00	0.00
$V_{max,adh}$	-0.46	-0.62	-0.50	-0.46
$K_{m,adh,ACALD}$	-0.35	-0.49	-0.38	-0.35
$K_{m,adh,ETOH}$	0.36	0.49	0.39	0.35
$K_{m,adh,NADH}$	0.35	0.48	0.37	0.35
$K_{m,adh,NAD}$	0.00	0.00	0.00	0.00
$K_{i,adh,ACALD}$	-0.09	-0.13	-0.10	-0.09
$K_{i,adh,ETOH}$	0.00	0.00	0.00	0.00
$K_{i,adh,NADH}$	-0.35	-0.49	-0.38	-0.35
$K_{i,adh,NAD}$	0.13	0.18	0.14	0.13
$K_{eq,adh}$	0.50	0.65	0.53	0.48
K_{suc}	0.02	0.00	0.02	0.00
K_{ace}	0.06	0.04	0.05	0.04
ATP	0.32	0.15	0.47	0.12
ADP	-0.26	-0.12	-0.40	-0.11
AMP	0.00	0.00	0.00	0.01
GLCo	0.19	1.04	0.11	0.45
CO2	0.00	0.00	0.00	0.00
ETOH	0.14	0.16	0.15	0.13
T6P	-1.36	-0.38	-1.34	-0.35
TREH	0.00	0.00	0.00	0.00
F26BP	0.00	0.00	0.00	0.00
GLY	0.00	0.00	0.00	0.00
ACE	0.00	0.00	0.00	0.00
