# Supplementary material for: Testing Biochemistry Revisited: How In Vivo Metabolism Can Be Understood from In Vitro Enzyme Kinetics
Source: PLoS Comput Biol. 2012 Apr 26;8(4):e1002483. doi: 10.1371/journal.pcbi.1002483 (PMC3343101; doi:10.1371/journal.pcbi.1002483)
Supplement: Table S15 — Flux Control Coefficients, D = 0.35 h−1, 4 h nitrogen-starved. (PDF) [file pcbi.1002483.s015.pdf]

Table S15 Flux Control Coefficients, D = 0.35 h^{-1}, 4h nitrogen-starved.

The control of the flux J through an enzyme j (rows) exerted by an enzyme i (columns). The control coefficient

$C_i^j = \frac{dJ_j}{dV_{\max,i}} \cdot \frac{V_{\max,i}}{J_j} = \frac{d\ln J_j}{d\ln V_{\max,i}} \approx \frac{\ln J_{j,+h} - \ln J_{j,0}}{\ln V_{\max,i,+h} - \ln V_{\max,i,0}}$ was approximated by increasing the V_{max} of the enzyme of interest by h, which was 1%. This was done for the model versions corresponding to figure 2 in the main text.

**Enzyme**	J_{glt}	J_{hxk}	J_{pgi}	J_{pfk}	J_{ald}	J_{gapdh}	J_{pgk}	J_{gpm}	J_{eno}	J_{pyk}	J_{pdc}	J_{adh}
GLT	0.56	0.53	0.53	0.53	0.53	0.62	0.62	0.62	0.62	0.62	0.62	0.67
HXK	0.13	0.13	0.13	0.13	0.13	0.15	0.15	0.15	0.15	0.15	0.15	0.16
PGI	0.00	0.00	0.00	0.00	0.00	0.00	0.00	0.00	0.00	0.00	0.00	0.00
PFK	0.12	0.12	0.12	0.12	0.12	0.13	0.13	0.13	0.13	0.13	0.13	0.15
ALD	0.00	0.00	0.00	0.00	0.00	0.00	0.00	0.00	0.00	0.00	0.00	0.00
GAPDH	0.00	0.00	0.00	0.00	0.00	0.00	0.00	0.00	0.00	0.00	0.00	0.00
PGK	0.00	0.00	0.00	0.00	0.00	0.00	0.00	0.00	0.00	0.00	0.00	0.00
GPM	0.00	0.00	0.00	0.00	0.00	0.00	0.00	0.00	0.00	0.00	0.00	0.00
ENO	0.00	0.00	0.00	0.00	0.00	0.00	0.00	0.00	0.00	0.00	0.00	0.00
PYK	0.00	0.00	0.00	0.00	0.00	0.00	0.00	0.00	0.00	0.00	0.00	0.00
PDC	0.00	0.00	0.00	0.00	0.00	0.00	0.00	0.00	0.00	0.00	0.00	0.00
ADH	0.00	0.00	0.00	0.00	0.00	0.00	0.00	0.00	0.00	0.00	0.00	0.00
