# Supplementary material for: Testing Biochemistry Revisited: How In Vivo Metabolism Can Be Understood from In Vitro Enzyme Kinetics
Source: PLoS Comput Biol. 2012 Apr 26;8(4):e1002483. doi: 10.1371/journal.pcbi.1002483 (PMC3343101; doi:10.1371/journal.pcbi.1002483)
Supplement: Table S16 — Comparison of the regulation coefficients presented in [23] ( Vmax values measured under enzyme-optimized conditions) and those re-calculated with the Vmax values measured under in vivo -like conditions, but using the same changes in flux. (PDF) [file pcbi.1002483.s016.pdf]

Table S16 Comparison of the regulation coefficients presented in [1] (V_{max} values measured under enzyme-optimized conditions) and those re-calculated with the V_{max} values measured under *in vivo*-like conditions, but using the same changes in flux. Glucose transport was assessed in whole cells in growth medium, hence only under physiological conditions. The hierarchical regulation coefficient ρ_h reflects regulation of the flux by altered V_{max} through the entire gene-expression cascade. The metabolic regulation coefficient ρ_m reflects regulation through interaction of the enzyme with altered metabolite concentrations. For theory see [2,3].

Enzyme	Chapter 3 Enzyme-optimized V_{max} values						*In vivo*-like V_{max} values					
	D = 0.1 h^{-1}			D = 0.35 h^{-1}			D = 0.1 h^{-1}			D = 0.35 h^{-1}		
	ρ_h	SEM	ρ_m	ρ_h	SEM	ρ_m	ρ_h	SEM	ρ_m	ρ_h	SEM	ρ_m
HXT							-1.5		2.5	2.0		-1.0
HXK	-1.0	0.3	2.0	0.3	0.2	0.7	-0.6	0.0	1.6	0.3	0.1	0.7
PGI	-0.2	0.2	1.2	0.1	0.3	0.9	0.1	0.1	0.9	0.4	0.1	0.6
PFK	-0.7	0.2	1.7	1.9	0.3	-0.9	-0.7	0.01	1.7	4.4	1.1	-3.4
ALD	-0.8	0.2	1.8	3.5	0.8	-2.5	-0.6	0.5	1.6	1.4	0.2	-0.4
GAPDH	-0.5	0.2	1.5	-0.2	-0.4	1.2	-0.5	0.3	1.5	0.3	0.5	0.7
PGK	0.2	0.3	0.8	1.1	0.3	-0.1	0.3	0.1	0.7	1.2	0.0	-0.2
PYK	-0.1	0.2	1.1	1.3	0.1	-0.3	0.4	0.2	0.6	2.0	0.2	-1.0
PDC	1.2	0.3	-0.2	6.6	1.9	-5.6	0.5	0.1	0.5	3.8	0.1	-2.8
ADH	-0.7	0.1	1.7	2.3	0.4	-1.3	-0.2	0.0	1.2	0.8	0.1	0.2

References

1. van Eunen K, Dool P, Canelas AB, Kiewiet J, Bouwman J, et al. (2010) Time-dependent regulation of yeast glycolysis upon nitrogen starvation depends on cell history. IET Syst Biol 4: 157-168.
2. Rossell S, van der Weijden CC, Lindenbergh A, van Tuijl A, Francke C, et al. (2006) Unraveling the complexity of flux regulation: a new method demonstrated for nutrient starvation in Saccharomyces cerevisiae. Proc Natl Acad Sci U S A 103: 2166-2171.
3. ter Kuile BH, Westerhoff HV (2001) Transcriptome meets metabolome: hierarchical and metabolic regulation of the glycolytic pathway. FEBS Lett 500: 169-171.
