# Supplementary material for: Testing Biochemistry Revisited: How In Vivo Metabolism Can Be Understood from In Vitro Enzyme Kinetics
Source: PLoS Comput Biol. 2012 Apr 26;8(4):e1002483. doi: 10.1371/journal.pcbi.1002483 (PMC3343101; doi:10.1371/journal.pcbi.1002483)
Supplement: Text S1 — Concentrations of substrates and coupling enzymes in the in vivo -like assays for each individual enzyme. (PDF) [file pcbi.1002483.s017.pdf]

Text S1 Concentrations of substrates and coupling enzymes in the *in vivo*-like assays for each individual enzyme

Hexokinase (HXK, EC 2.7.1.1) – 1 mM NADP, 10 mM glucose, 1 mM ATP and 1.8 U/ml glucose-6-phosphate dehydrogenase (G6PDH, EC 1.1.1.49).

Phosphoglucose isomerase (PGI, EC 5.3.1.9, reverse direction) – 0.4 mM NADP, 2 mM Fructose 6-phosphate (F6P) and 1.8 U G6PDH.

Phosphofructokinase (PFK, EC 2.7.1.11) – 0.1 mM fructose 2,6-bisphosphate, 0.15 mM NADH, 0.5 mM ATP, 10 mM F6P, 0.45 U/ml aldolase (ALD, EC 4.1.2.13), 0.6 U/ml Glycerol 3-phosphate dehydrogenase (G3PDH, EC 1.1.1.8) and 1.8 U/ml triosephosphate isomerase (TPI, EC 5.3.1.1).

Aldolase – 0.15 mM NADH, 2 mM fructose 1,6-bisphosphate (F16BP) , 0.6 U/ml G3PDH and 1.8 U/ml TPI.

Glyceraldehyde 3-phosphate dehydrogenase (GAPDH, EC 1.2.1.12, reverse direction) – 1 mM ATP, 0.15 mM NADH, 5 mM 3-phosphoglyceric acid (3PG) and 22.5 U/ml 3-phosphoglycerate kinase (PGK, EC 2.7.2.3).

Glyceraldehyde 3-phosphate dehydrogenase (GAPDH, forward direction) – 10 mM ADP, 1 mM NAD, 5.8 mM glyceraldehyde 3-phosphate and 22.5 U/ml PGK.

3-phosphoglycerate kinase (reverse direction) – 1 mM ATP, 0.15 mM NADH, 5 mM 3PG and 8 U/ml GAPDH.

Phosphoglycerate-mutase (GPM, EC 5.4.2.1) – 10 mM ADP, 0.15 mM NADH, 1.25 mM 2,3-diphospho-D-glyceric acid, 5 mM 3PG, 2 U/ml enolase (ENO, EC 4.2.1.11), 13 U/ml pyruvate kinase (PYK, EC 2.7.1.40) and 11.3 U/ml lactate dehydrogenase (LDH, EC 1.1.1.27).

Enolase – The activity of ENO was measured by the production rate of PEP, which was analyzed with a spectrophotometer using a wavelength of 240 nm. The assay was measured using the *in vivo*-like assay medium with 6 mM of 2-phosphoglyceric acid (2PG).

Pyruvate kinase – 10 mM ADP, 0.15 mM NADH, 1 mM F16BP, 2 mM phosphoenolpyruvate (PEP) and 13.8 U/ml LDH.

Pyruvate decarboxylase (PDC, EC 4.1.1.1) – 0.2 mM TPP, 0.15 mM NADH, 50 mM pyruvate and 88 U/ml alcohol dehydrogenase (ADH, EC 1.1.1.1).

Alcohol dehydrogenase – 1 mM NAD and 100 mM ethanol.

The affinity constant (K_m) of GAPDH for NAD was redetermined in the *in vivo*-like assay medium by varying the substrate concentrations.
