# Supplementary material for: Testing Biochemistry Revisited: How In Vivo Metabolism Can Be Understood from In Vitro Enzyme Kinetics
Source: PLoS Comput Biol. 2012 Apr 26;8(4):e1002483. doi: 10.1371/journal.pcbi.1002483 (PMC3343101; doi:10.1371/journal.pcbi.1002483)
Supplement: Text S2 — Ordinary differential equations. (PDF) [file pcbi.1002483.s018.pdf]

Text S2 Ordinary differential equations

$$\frac{dGlc_i}{dt} = v_{glt} - v_{hk} + 2 \cdot v_{tre1} \quad (1)$$

$$\frac{dG6P}{dt} = v_{hk} - v_{pgi} - 2 \cdot v_{tre2} \quad (2)$$

$$\frac{dF6P}{dt} = v_{pgi} - v_{pfk} \quad (3)$$

$$\frac{dF16P}{dt} = v_{pfk} - v_{ald} \quad (4)$$

$$\frac{dTRIO}{dt} = 2 \cdot v_{ald} - v_{gly} - v_{gapdh} \quad (5)$$

$$\frac{dBPG}{dt} = v_{gapdh} - v_{pgk} \quad (6)$$

$$\frac{dP3G}{dt} = v_{pgk} - v_{gpm} \quad (7)$$

$$\frac{dP2G}{dt} = v_{gpm} - v_{eno} \quad (8)$$

$$\frac{dPEP}{dt} = v_{eno} - v_{pyk} \quad (9)$$

$$\frac{dPYR}{dt} = v_{pyk} - v_{pdc} - 2 \cdot v_{suc} \quad (10)$$

$$\frac{dACALD}{dt} = v_{pdc} - v_{adh} - v_{ace} \quad (11)$$

$$\frac{dNADH}{dt} = -v_{gly} + v_{gapdh} - v_{adh} + 3 \cdot v_{suc} + v_{ace} \quad (12)$$
