# Supplementary material for: Testing Biochemistry Revisited: How In Vivo Metabolism Can Be Understood from In Vitro Enzyme Kinetics
Source: PLoS Comput Biol. 2012 Apr 26;8(4):e1002483. doi: 10.1371/journal.pcbi.1002483 (PMC3343101; doi:10.1371/journal.pcbi.1002483)
Supplement: Text S3 — Rate equations. (PDF) [file pcbi.1002483.s019.pdf]

Text S3 Rate equations

$$v_{glt} = \frac{V_{\max,glt} \cdot \left(\frac{Glc_o}{K_{m,glt,Glc}} - \frac{Glc_i(t)}{K_{m,glt,Glc} \cdot K_{eq,glt}} \right)}{1 + \frac{Glc_o}{K_{m,glt,Glc}} + \frac{Glc_i(t)}{K_{m,glt,Glc}} + P \cdot \frac{Glc_o \cdot Glc_i(t)}{K_{m,glt,Glc} \cdot K_{m,glt,Glc}}} \tag{13}$$

$$v_{hk} = \frac{V_{\max,hk} \cdot \left(\frac{Glc_i(t)}{K_{m,hk,Glci}} \cdot \frac{ATP}{K_{m.hk,ATP}} - \frac{G6P(t) \cdot ADP}{K_{m,hk,Glci} \cdot K_{m,hk,ATP} \cdot K_{eq,hk}} \right)}{\left(1 + \frac{Glc_i(t)}{K_{m,hk,Glci}} + \frac{G6P(t)}{K_{m,hk,G6P}} + \frac{T6P}{K_{i,hk,T6P}} \right) \cdot \left(1 + \frac{ATP}{K_{m,hk,ATP}} + \frac{ADP}{K_{m,hk,ADP}} \right)} \tag{14}$$

$$v_{tre1} = K_{tre1} \tag{15}$$

$$v_{tre2} = K_{tre2} \tag{16}$$

$$v_{pgi} = \frac{V_{\max,pgi} \cdot \left(\frac{G6P(t)}{K_{m,pgi,G6P}} - \frac{F6P(t)}{K_{m,pgi,F6P} \cdot K_{eq,pgi}} \right)}{1 + \frac{G6P(t)}{K_{m,pgi,G6P}} + \frac{F6P(t)}{K_{m,pgi,F6P}}} \tag{17}$$

$$v_{pfk} = \frac{V_{\max,pfk} \cdot gR_{PFK} \cdot F6P(t) \cdot ATP \cdot \left(1 + \frac{F6P(t) \cdot ATP}{K_{m,pfk,F6P} \cdot K_{m,pfk,ATP}} + \frac{gR_{PFK} \cdot F6P(t) \cdot ATP}{K_{m,pfk,F6P} \cdot K_{m,pfk,ATP}} \right)}{K_{m,pfk,F6P} \cdot K_{m,pfk,ATP} \left(\left(1 + \frac{F6P(t) \cdot ATP}{K_{m,pfk,F6P} \cdot K_{m,pfk,ATP}} + \frac{gR_{PFK} \cdot F6P(t) \cdot ATP}{K_{m,pfk,F6P} \cdot K_{m,pfk,ATP}} \right)^2 + L_{PFK} \cdot \left(1 + \frac{C_{pfk,ATP} \cdot ATP}{K_{m,pfk,ATP}} \right)^2 \right)} \tag{18}$$

$$L_{PFK} = L_{0,PFK} \left(\frac{1 + \frac{C_{i,pfk,ATP} \cdot ATP}{K_{pfk,ATP}}}{1 + \frac{ATP}{K_{pfk,ATP}}} \right)^2 \cdot \left(\frac{1 + \frac{C_{i,pfk,AMP} \cdot AMP}{K_{pfk,AMP}}}{1 + \frac{AMP}{K_{pfk,AMP}}} \right)^2 \cdot \left(\frac{1 + \frac{C_{i,pfk,F26BP} \cdot F26BP}{K_{pfk,F26BP}} + \frac{C_{i,pfk,F16BP} \cdot F16BP}{K_{pfk,F16BP}}}{1 + \frac{F26BP}{K_{pfk,F26BP}} + \frac{F16BP}{K_{pfk,F16BP}}} \right)^2$$

$$v_{ald} = \frac{V_{\max,ald} \cdot \left(\frac{F16BP(t)}{K_{m,ald,F16BP}} - \frac{\frac{K_{eq,TPI} \cdot TRIO(t)}{1 + K_{eq,TPI}} \cdot \frac{TRIO(t)}{1 + K_{eq,TPI}}}{K_{m,ald,F16BP} \cdot K_{eq,ald}} \right)}{1 + \frac{F16BP(t)}{K_{m,ald,F16BP}} + \frac{\frac{K_{eq,TPI} \cdot TRIO(t)}{1 + K_{eq,TPI}}}{K_{m,ald,GAP}} + \frac{\frac{TRIO(t)}{1 + K_{eq,TPI}}}{K_{m,ald,DHAP}} + \frac{\frac{K_{eq,TPI} \cdot TRIO(t)}{1 + K_{eq,TPI}} \cdot \frac{TRIO(t)}{1 + K_{eq,TPI}}}{K_{m,ald,GAP} \cdot K_{m,ald,DHAP}} + \frac{F16P(t) \cdot \frac{K_{eq,TPI} \cdot TRIO(t)}{1 + K_{eq,TPI}}}{K_{i,ald,GAP} \cdot K_{m,ald,F16BP}}} \tag{19}$$

$$v_{gly} = K_{gly} \tag{20}$$

$$v_{gapdh} = \frac{\frac{V^+_{\max,gapdh} \cdot \frac{K_{eq,TPI} \cdot TRIO(t)}{1 + K_{eq,TPI}} \cdot (NADt - NADH(t))}{K_{m,gapdh,GAP} \cdot K_{m,gapdh,NAD}} - \frac{V^-_{\max,gapdh} \cdot BPG(t) \cdot NADH(t)}{K_{m,gapdh,BPG} \cdot K_{m,gapdh,NADH}}}{\left(1 + \frac{\frac{K_{eq,TPI} \cdot TRIO(t)}{1 + K_{eq,TPI}}}{K_{m,gapdh,GAP}} + \frac{BPG(t)}{K_{m,gapdh,BPG}} \right) \cdot \left(1 + \frac{NADt - NADH(t)}{K_{m,gapdh,NAD}} + \frac{NADH(t)}{K_{m,gapdh,NADH}} \right)} \tag{21}$$

$$v_{pgk} = \frac{V^-_{\max,pgk} \cdot \left(\frac{K_{eq,gpk} \cdot BPG(t) \cdot ADP}{K_{m.pgk,BPG} \cdot K_{m.pgk,ATP}} - \frac{P3G(t) \cdot ATP}{K_{m.pgk,P3G} \cdot K_{m.pgk,ATP}} \right)}{\left(1 + \frac{BPG(t)}{K_{m.pgk,BPG}} + \frac{P3G(t)}{K_{m.pgk,P3G}} \right) \cdot \left(1 + \frac{ATP}{K_{m.pgk,ATP}} + \frac{ADP}{K_{m.pgk,ADP}} \right)} \tag{22}$$

$$v_{gpm} = \frac{V_{\max,gpm} \cdot \left(\frac{P3G(t)}{K_{m,gpm,P3G}} - \frac{P2G(t)}{K_{m,gpm,P2G} \cdot K_{eq,gpm}} \right)}{1 + \frac{P3G(t)}{K_{m,gpm,P3G}} + \frac{P2G(t)}{K_{m,gpm,P2G}}} \tag{23}$$

$$v_{eno} = \frac{V_{\max,eno} \cdot \left(\frac{P2G(t)}{K_{m,eno,P2G}} - \frac{PEP(t)}{K_{m,eno,PEP} \cdot K_{eq,eno}} \right)}{1 + \frac{P2G(t)}{K_{m,eno,P2G}} + \frac{PEP(t)}{K_{m,eno,PEP}}} \tag{24}$$

$$v_{pyk} = \frac{V_{\max,pyk} \cdot \frac{PEP(t)}{K_{m,pyk,PEP}} \cdot \left(\frac{PEP(t)}{K_{m,pyk,PEP}} + 1 \right)^{n_{pyk}-1}}{L_{0,pyk} \cdot \left(\frac{\frac{ATP}{K_{m,pyk,ATP}} + 1}{\frac{F16P(t)}{K_{m,pyk,F16P}} + 1} \right)^{n_{pyk}} + \left(1 + \frac{PEP(t)}{K_{m,pyk,PEP}} \right)^{n_{pyk}}} \cdot \frac{ADP}{ADP + K_{m,pyk,ADP}} \tag{25}$$

$$v_{pdc} = \frac{V_{\max,pdc} \cdot PYR(t)^{NH_{PDC}}}{K_{m,pdc,PYR} \cdot \left(1 + \frac{PYR(t)^{NH_{PDC}}}{K_{m,pdc,PYR}{}^{NH_{PDC}}} \right)} \tag{26}$$

$$v_{adh} = -\frac{V^{-}_{\max,adh} \cdot \left(\frac{(NADt - NADH(t)) \cdot ETOH}{K_{i,adh,NAD} \cdot K_{m,adh,ETOH}} - \frac{NADH(t) \cdot ACALD(t)}{K_{i,adh,NAD} \cdot K_{m,adh,ETOH} \cdot K_{eq,adh}} \right)}{\left(\begin{array}{c} 1 + \frac{NADt - NADH(t)}{K_{i,adh,NAD}} + \frac{K_{m,adh,NAD} \cdot ETOH}{K_{i,adh,NAD} \cdot K_{m,adh,ETOH}} + \frac{K_{m,adh,NADH} \cdot ACALD(t)}{K_{i,adh,NAD} \cdot K_{m,adh,ACALD}} \\ + \frac{NADH(t)}{K_{i,adh,NADH}} + \frac{(NADt - NADH(t)) \cdot ETOH}{K_{i,adh,NAD} \cdot K_{m,adh,ETOH}} + \frac{K_{m,adh,NADH} \cdot (NADt - NADH(t)) \cdot ACALD(t)}{K_{i,adh,NAD} \cdot K_{i,adh,NADH} \cdot K_{m,adh,ACALD}} \\ + \frac{K_{m,adh,NAD} \cdot NADH(t) \cdot ETOH}{K_{i,adh,NAD} \cdot K_{i,adh,NADH} \cdot K_{m,adh,ETOH}} + \frac{NADH(t) \cdot ACALD(t)}{K_{i,adh,NADH} \cdot K_{m,adh,ACALD}} \\ + \frac{(NADt - NADH(t)) \cdot ETOH \cdot ACALD(t)}{K_{i,adh,NAD} \cdot K_{m,adh,ETOG} \cdot K_{i,adh,ACALD}} + \frac{NADH(t) \cdot ETOH \cdot ACALD(t)}{K_{i,adh,ETOH} \cdot K_{i,adh,NADH} \cdot K_{m,adh,ACALD}} \end{array} \right)} \tag{27}$$

$$v_{suc} = K_{suc} \tag{28}$$

$$v_{ace} = K_{ace} \cdot ACALD(t) \tag{29}$$
